# Platelets in Healthy and Disease States: From Biomarkers Discovery to Drug Targets Identification by Proteomics

**DOI:** 10.3390/ijms21124541

**Published:** 2020-06-25

**Authors:** Erica Gianazza, Maura Brioschi, Roberta Baetta, Alice Mallia, Cristina Banfi, Elena Tremoli

**Affiliations:** Proteomics Unit, Monzino Cardiologic Center IRCCS, 20138 Milan, Italy; erica.gianazza@ccfm.it (E.G.); maura.brioschi@ccfm.it (M.B.); roberta.baetta@ccfm.it (R.B.); alice.mallia@ccfm.it (A.M.); elena.tremoli@ccfm.it (E.T.)

**Keywords:** blood cells, proteins, mass spectrometry, antiplatelet drugs, post-translational modifications

## Abstract

Platelets are a heterogeneous small anucleate blood cell population with a central role both in physiological haemostasis and in pathological states, spanning from thrombosis to inflammation, and cancer. Recent advances in proteomic studies provided additional important information concerning the platelet biology and the response of platelets to several pathophysiological pathways. Platelets circulate systemically and can be easily isolated from human samples, making proteomic application very interesting for characterizing the complexity of platelet functions in health and disease as well as for identifying and quantifying potential platelet proteins as biomarkers and novel antiplatelet therapeutic targets. To date, the highly dynamic protein content of platelets has been studied in resting and activated platelets, and several subproteomes have been characterized including platelet-derived microparticles, platelet granules, platelet releasates, platelet membrane proteins, and specific platelet post-translational modifications. In this review, a critical overview is provided on principal platelet proteomic studies focused on platelet biology from signaling to granules content, platelet proteome changes in several diseases, and the impact of drugs on platelet functions. Moreover, recent advances in quantitative platelet proteomics are discussed, emphasizing the importance of targeted quantification methods for more precise, robust and accurate quantification of selected proteins, which might be used as biomarkers for disease diagnosis, prognosis and therapy, and their strong clinical impact in the near future.

## 1. Platelet Biology and its Roles in Human Diseases

### 1.1. Introduction

#### 1.1.1. Platelet biology: An Overview

Platelets are small anucleate blood cells produced by megakaryocytes in the bone marrow and lungs [1,2]. Once released by their megakaryocytic precursor, platelets enter the bloodstream and circulate for 7–10 days, after which they are cleared in the spleen and liver [3]. Platelets are highly specialized effector cells in physiological haemostasis and play a central role in pathological thrombosis [3]. In primary haemostasis, they rapidly adhere to the damaged vessel wall at the site of injury and aggregate to form a platelet plug. Failure to form an adequate plug underlies bleeding disorders, while excessive platelet reactivity leads to an increased risk of thrombosis. 

Platelets are now known to play major effector activities in a number of additional functions, including inflammatory reactions and innate immune responses [4]. Instrumental to these activities is the ability of platelets to respond to signals from the endothelium, circulating cells, or other blood components [3]. Platelets are present in high numbers in the circulation (150.000 to 400.000 per microliter of whole blood in humans) and they continuously patrol their environment using cell surface receptors and adhesion molecules, including integrins, selectins, toll-like receptors, transmembrane receptors, immunoglobulin superfamily receptors, tyrosine kinase receptors, lipid receptors and others [5,6]. Moreover, platelets can alter the environment in response to various stimuli through the release of bioactive mediators from different storage granules (α-granules, dense granules and lysosomes), bioactive lipid products formed by oxidation of free fatty acids, and extracellular vesicles [3,7,8,9]. The secretion products, including coagulation factors, growth factors, chemokines, cytokines, microbicidal proteins, prostaglandins, thromboxane A2 (TXA2), eicosanoids, and RNA species, influence many physiological and pathophysiological processes beyond haemostasis [3,5,7,10].

Platelet activation includes numerous signaling pathways, through local prothrombotic factors and platelet secretion products. Platelet adhesion to the extracellular matrix involves the binding between exposed collagen and platelet glycoprotein receptors, causing the platelet shape to change and the release of their granules contents.

Platelets are not a homogeneous cell population and their morphological heterogeneity is present at rest, upon stimulation by agonists, and within the haemostatic plug. Circulating platelets are heterogeneous in size, age, and responsiveness [11]. Studies investigating the functional differences of platelet subpopulations have emerged only in the second half of the last century [3,11]. However, the causes of platelet functional heterogeneity and how structural heterogeneity relates to variation in platelet responses remain largely unknown [3].

Although platelets are anucleate, they have long been known to contain RNAs. This genomic material was not merely a remnant from the precursor megakaryocyte, but rather the result of a highly regulated sorting process by which megakaryocytes invest platelets with mRNA during thrombopoiesis [12]. Platelets display a diverse repertoire of coding and noncoding RNAs, diverse pathways for processing RNA transcripts, specialized mechanisms of translation, and the capacity to synthesize new proteins and alter the constitutive platelet proteome in response to activating signals [13]. In addition, differential transfer of RNAs from megakaryocyte to platelet, and from various circulating cells, can alter gene expression in platelets [11]. Therefore, platelets have high versatility and adaptability in structure and function, and the platelet transcriptome has a relevant role in mediating platelet function in health and disease [3,13,14]. Multiple mutations associated with defects of platelet function have been identified in genes encoding receptors, intracellular signaling proteins, cytoskeletal proteins, and proteins regulating the biogenesis of platelet granules [3,15,16].

Genomics and transcriptomics of megakaryocytes and platelets are now being extensively investigated in basic and clinical studies. Moreover, because platelets circulate systemically and are easily obtained, several recent studies have highlighted the potential use of platelet transcripts as biomarkers, even for diseases without apparent platelet etiology [12].

Over the years, proteomic studies provided additional important information concerning the platelet biology and their related diseases [17]. Recent advances helped to elucidate platelet localization, interactions, post-translational modifications (PTMs), and activation states of gene products. The highly dynamic protein content of platelets has been studied using resting and activated platelets, and various subproteomes have been characterized including releasates, granules, platelet-derived microparticles (PMPs), membrane and cytoskeletal proteins, and specific PTMs occurring in platelets.

#### 1.1.2. Platelets and Proteomics

Platelet dysfunction is often attributable to alterations in protein expression and dynamic occurrence of PTMs. Therefore, particular attention must be paid to studying their proteome in order to understand better their biological mechanisms and multiple functions. Platelets can be easily isolated from the human samples and show limited levels of protein synthesis, making the proteomic application very interesting. Proteomics has emerged as a powerful tool for characterizing the complexity of platelet functions in health and disease as well as for identifying potential novel antiplatelet therapeutic targets [18]. Therefore, proteomics, in combination with the other “omic” technologies, may contribute to improving the knowledge of complex processes underlying the platelet response to several pathophysiological pathways.

Over the past twenty years, mass spectrometry (MS) and its application in proteomic studies lead to the compilation of an extensive list of proteins expressed in platelets and relevant data on protein–protein interactions and PTMs. Novel and more sensitive MS-based instruments and technologies provided the possibility to identify and quantify ever lower protein amounts. Numerous studies demonstrated the ability of proteomics to measure the differences of proteins and their isoforms quantitatively, covering about 6–7 logs of dynamic range in abundance.

Mass spectrometry ensures high-sensitivity, specific and throughput analysis of a given proteome and high-performance liquid chromatography (LC) systems before the MS analysis lead to a significant increase in the separation of highly complex samples. Furthermore, thanks to quantitative MS measurements, either based on label-free approaches or stable isotope labelling, considerable improvements have been made in the large-scale analysis of proteins and their modifications.

In this review, we provide a critical overview on the most important platelet proteomic studies, in healthy and disease conditions, dedicated to the different aspects of platelet biology from signaling to granules content and the impact of drugs on platelet functions. Recent advances in quantitative platelet proteomics are crucial for better understanding platelet activation and aggregation processes, and they will have a strong clinical impact in the near future [19].

### 1.2. Platelet Activation and Signaling

#### 1.2.1. Proteins Involved in Platelet Activation and Signaling

Platelet activation is a complex biological process that includes numerous signaling pathways. Cytoskeletal and signaling proteins have an important role in platelet functions, and thus they are crucial targets for the platelet proteomic studies.

Platelet adhesion to the extracellular matrix involves the binding between exposed collagen and platelet glycoprotein receptors, causing the platelet shape to change and the release of their granules contents. Platelets contain at least three major granules types and granule exocytosis has a fundamental role in platelet activities [20]. Multiple pathways can stimulate platelet activation, through local prothrombotic factors (e.g., tissue factor (TF)) and platelet secretion products. Glycoprotein VI (GPVI) is the primary signaling receptor on platelet membranes involved in their activation on exposed collagen [21,22]. Platelet activation by the GPVI receptor is mediated by immunoreceptor tyrosine-based activation motif signaling, but also G-protein-coupled receptor-mediated signaling can influence platelet response to several soluble agonists such as adenosine diphosphate (ADP), serotonin, TXA2, prostaglandin E2 (PGE2) and thrombin [23]. These molecules increase the response to injury, promoting an extended platelet aggregation, and between them, thrombin is the most potent agonist able to activate platelets mainly through the interaction with protease-activated receptors (PAR) on their surface. Thrombin activates both PAR-1 and PAR-4 on human platelets with distinct mechanisms that have important implications for the development of PAR antagonists preventing thrombin-induced platelet activation [24]. The final event of platelet activation is the upregulation of integrin adhesion receptors, among which the most important is Glycoprotein IIb/IIIa (GPIIb/IIIa) receptor that allows the binding of fibrinogen or von Willebrand factor (vWF), contributing to platelet aggregation. Activated platelets interact with the vascular endothelium and circulating leukocytes through P-selectin, and play a central role in inflammation, thrombosis, and atherogenesis [25,26]. Moreover, activated platelets express functional CD40 ligand (CD40L) (also known as CD154), which is a transmembrane molecule involved in cell signaling in innate and adaptive immunity [27]. The CD40L also binds receptor CD40 expressed on endothelial cells to stimulate the secretion of chemokines and synthesis of adhesion molecules. The CD40L may be cleaved and released in its soluble form (sCD40L), which is produced by platelets only after activation and has a cytokine-like role, inducing the expression of E-selectin, P-selectin and vascular cell adhesion molecule 1 (VCAM-1) on vascular cells, as well as the release of matrix metalloproteinases (MMPs) and interleukin 6 and 8 (IL-6 and IL-8) [27]. The sCD40L is clearly involved in the pathophysiology of atherosclerosis and atherothrombosis [28,29].

**Figure 1:**
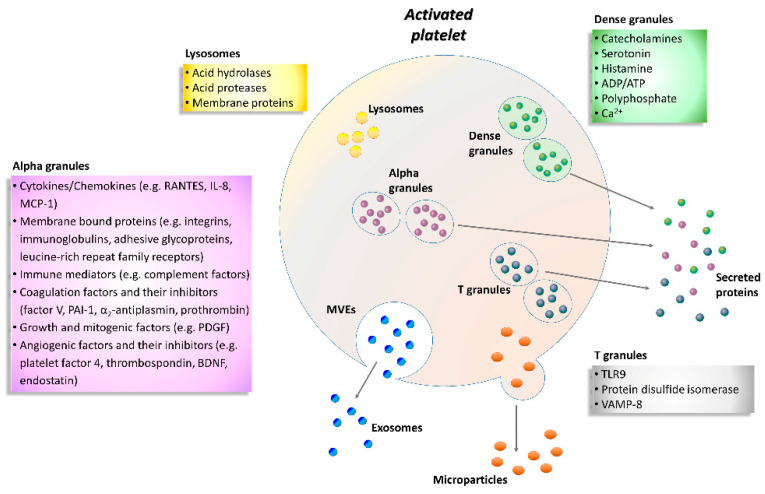
Schematic representation of the activated platelet showing multivesicular elements, exosomes, microparticles and specific granule releasates. Relevant proteins and factors are released following platelet activation. A plethora of biological substances including adhesion molecules, cytokines, chemokines, coagulation factors, angiogenic factors, immunologic mediators, growth factors, and lysosomal enzymes are grouped according to the type of granule. ADP, adenosine diphosphate; ATP, adenosine triphosphate; BDNF, brain-derived neurotrophic factor; IL-8, interleukin-8; MCP-1, monocyte chemotactic protein-1; MVEs, multivesicular elements; PAI-1, plasminogen activator inhibitor-1; PDGF, platelet-derived growth factor; RANTES, regulated on activation, normal T cell expressed and secreted; TLR9, toll-like receptor 9; VAMP-8, vesicle-associated membrane protein-8.

Platelet factor 4 (PF4) or CXC motif chemokine ligand 4 is produced by platelets and it is contained in their α-granules (Figure 1). Upon platelet activation, PF4 is released into the circulation, where it covers several important roles in physiological and pathological conditions. It is involved in both inflammation and angiogenesis in wound healing through regulation of growth factor activity and platelet activation [30]. In addition, PF4 promotes reactive oxygen species (ROS) generation in vascular disorders [31], such as atherosclerosis or ischemia/reperfusion injury, and monocyte recruitment to the endothelium by means of its binding with RANTES (regulated on activation, normal T cell expressed and secreted), which is stored in high amounts in platelet α-granules and plays an active role in the atherosclerotic disease process [32]. Both PF4 and RANTES are released from α-granules upon platelet activation and hetero-aggregates of PF4 and RANTES promote monocyte adhesion in inflammation or atherosclerosis [33]. 

Besides their central role in haemostasis, platelets also interact with and remove pathogens from the blood stream at sites of infection and inflammation [34]. Platelets bind activated neutrophils, inducing the formation of neutrophil extracellular traps (NETs), which contain externalized DNA and DNA-associated nuclear and granular proteins, such as neutrophil elastase and myeloperoxidase. NETs can also acquire TF from the blood and can entrap platelets and fibrin. Although physiologically beneficial when released during an infection, an uncontrolled and excessive NET formation may contribute to the initiation and progression of atherosclerotic lesions and to arterial, venous, and cancer-associated thrombosis [35]. Moreover, NETs are found in a variety of other conditions such as lung injury and autoimmune diseases.

Enzyme-linked immunosorbent assay (ELISA) or Western blot are commonly used to study platelets activation markers; however, flow cytometry is a more standardized method that allows quantification of the expression of platelet activation markers and receptors and their association with other blood cells [21]. Mainly three types of granules have been found inside the platelets: α-granules, dense granules, and lysosomes (Figure 1). In addition, T granules have been described as another platelet intracellular compartment that is characterized by the coexpression of toll-like receptor 9 and protein disulfide isomerase during pro-platelet production [36]. All these granules are storage of adhesion molecules, cytokines, chemokines and coagulation or angiogenic factors (Figure 1).

In particular, α-granules are the most abundant type, about 50–80 per platelet that include both membrane-bound proteins and soluble proteins [37]. Membrane-bound proteins are then expressed on the platelet surface and comprise integrins, immunoglobulins, adhesive glycoproteins, leucine-rich repeat family receptors and other granule membrane-specific receptors. Hundreds of soluble proteins are typically released by α-granules and many of them are present in plasma with differences in structure or function. Secreted α-granule proteins are bioactive proteins involved in wide-ranging physiologic functions, among which innate immunity, inflammation, coagulation and mitogenesis, and with opposing activities (e.g., pro- and anticoagulants, proteases and inhibitors).

Dense granules have a distinct specific cargo regarding both biogenesis and function, which contains a few types of small molecules, such as catecholamines, ADP, ATP, polyphosphate and Ca^2+^ [38].

Instead, the third type of granule, platelet lysosome, contains primarily acid hydrolases and proteases [39]. Their secretion has a significant role in digestion of phagocytic and cytosolic components, as well as fibrinolysis and degradation of extracellular matrix (ECM) components, and vascular remodeling.

Activated platelets release numerous chemokines that control the movement of leukocytes from the vasculature towards the site of tissue damage or infection, regulate the phagocytosis and ROS production. Moreover, the high expression of adhesion molecules and ligands on the platelet surface promotes the interaction between platelets and endothelium or leukocytes, during haemostasis and inflammation. Integrins also facilitate platelets to bind to ECM molecules and other cells playing an important role in cell signaling. Since platelet surface markers have, in general, short detectability in human blood, platelet-monocyte aggregates have been detected in several studies as a more sensitive and accurate marker that describes platelet activation and a prothrombotic state in diseases, like advanced atherosclerosis, stable coronary artery disease, acute myocardial infarction, systemic inflammatory and autoimmune disorders, and neoplasms [40,41,42]. Similarly, circulating PMPs, that are the most abundant microparticles in the bloodstream (approximately 70–90% of circulating microparticles), are considered as potential biological markers for platelet activation [43]. They carry several unique proteins derived from platelets and mediate the communication between cells, promote the release of cytokines, and are involved in inflammation, angiogenesis, cancer progression and tissue regeneration [44].

Platelet microparticles have great relevance in a wide range of disease processes and high circulating levels have been observed in patients with cardiovascular diseases (CVDs), such as atherosclerosis [45], hypertension [46], thrombosis [47], and stroke [48]. Research interest in PMPs has grown past few years and focused on the development of more accurate methods for quantifying them to clarify their physiological role and their potential involvement in several clinical situations as disease biomarkers or new targets for antiplatelet drugs [44].

#### 1.2.2. Platelet Priming 

As mentioned before, within the bloodstream, platelets are subjected to the influence of a wide spectrum of activating and inactivating biomolecules and conditions [3]. In physiological states, the net result of these influences is the inhibition of spontaneous platelet adhesion and activation. In disease conditions, the threshold for platelet activation can be increased (negative platelet priming) or lowered (positive platelet priming) by systemic and local changes in the balance between activating and inactivating factors, resulting in altered responsiveness of platelets to agonists [3]. Negative platelet-priming substances include vessel wall-derived factors, such as nitric oxide, prostaglandin I2 (PGI2), adenosine, and thrombomodulin, and bioactive mediators present in plasma, such as PGE2, insulin, and polyunsaturated fatty acid products of 12-lipoxygenase. Positive priming factors include vessel wall-derived factors, such as vWF multimers and intercellular adhesion molecules, and bioactive mediators present in plasma, such as adrenaline, insulin-like growth factor 1, thrombopoietin, growth arrest-specific protein 6, sCD40L, cholesterol, PGE2 (at low-dose), and stromal cell-derived factor-1α [3]. 

The excitability of platelets has been shown to be altered in a number of conditions associated with high risk of developing atherosclerosis-related CVDs, such as hyperlipidemia [49], diabetes mellitus (DM) [50,51], hypertension [52], obesity [53] and cigarette-smoke exposure [54,55], as well as in other disease conditions including autoimmune diseases [56], hematological disorders and cancer [57]. Thus, modulation of platelet responsiveness due to platelet priming is likely to have important pathophysiological roles. However, whether platelet priming is a cause or consequence of the pathogenesis of diseases requires further investigation, since both acute and chronic disease conditions may themselves act as primers, either through exposure to tissue damage or through inflammatory mediators or pathogens [58].

### 1.3. The Role of Platelets in Disease 

**Figure 2:**
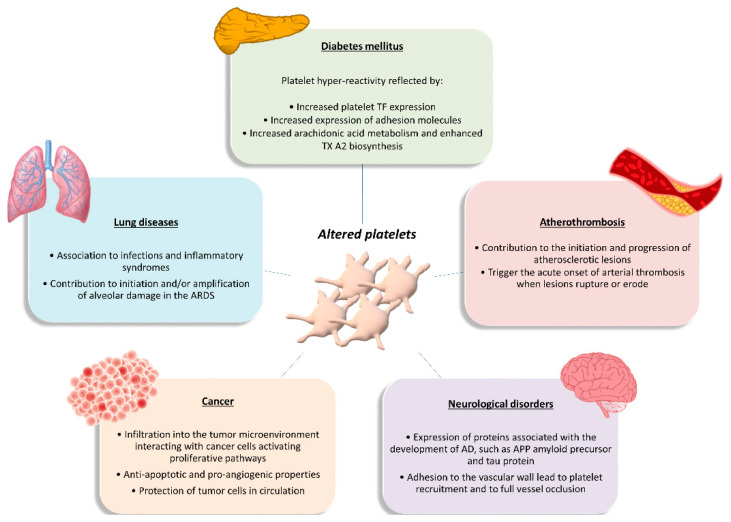
Functional role of altered platelets in the pathophysiology of several diseases. Brief overview of altered platelet contribution to atherothrombosis, diabetes mellitus, lung diseases, cancer, and neurological disorders. AD, Alzheimer’s disease; APP, amyloid precursor protein; ARDS, acute respiratory distress syndrome; TF, tissue factor; TXA2, thromboxane A2.

Developments in the field of platelet biology have led to new insights into platelet formation, function, heterogeneity, genetics, signaling and communication. The emergence of newly discovered and previously unrecognized biological capacities of platelets has led to the increasing recognition that platelets have a functional role in the pathophysiology of a wide variety of diseases, beyond the disorders of coagulation (Figure 2). The aim of this chapter is to provide a brief overview of the current understanding of platelet contribution to diseases not traditionally related to platelet number and/or function, for which platelet proteomics studies are available. Instead, this chapter will not address the role of platelets in hemostatic diseases. For an in-depth view of the recent progress in haemostatic diseases readers are referred to up-to-date publication [59].

#### 1.3.1. Platelets in Atherothrombosis

Platelets are essential for primary haemostasis and repair of the endothelium, but they also contribute to all stages of atherothrombosis [60]. Under physiological conditions, the intact, non-activated endothelium prevents platelet adhesion to the arterial wall. Under inflammatory conditions, however, platelets can adhere to the activated endothelial cell monolayer via adhesion receptors such as GPIb and GPVI and become activated [60,61]. Activated platelets release inflammatory and mitogenic mediators into the microenvironment, thereby altering the chemotactic, adhesive, and proteolytic properties of the endothelium. These platelet-induced alterations of endothelial-cell functions support leukocyte recruitment into nascent atherosclerotic plaques [60]. Moreover, platelets communicate biochemical signals to neutrophils, monocytes, and subsets of lymphocytes through adhesive molecules, such as P-selectin, and a multitude of secreted soluble mediators [62]. The initial contact is driven by the exposure of P-selectin on the activated platelets, which binds to P-selectin glycoprotein ligand 1 (PSGL-1) on the leukocyte surface, enhancing their adhesion on activated endothelial cells and inducing the production of TF by monocytes [60,62]. Signaling by P-selectin also stimulates monocytes and macrophages to produce chemoattractants or growth factors. Moreover, engagement by P-selectin of the PSGL-1 on the leukocyte surface initiates the formation of platelet-leukocyte aggregates that trigger mutual activation and release of bioactive mediators by both platelets and leukocytes, thereby modulating leukocyte function and fine-tuning immune responses [60,62,63]. Activated platelets also release chemokines that promote the differentiation of monocytes into macrophages (e.g., PF4), as well as matrix-degrading enzymes such as MMP 2 or 9 [60]. Moreover, activated platelets disseminate microparticles, which are intact vesicles that form by budding from the membrane. As with whole platelets, these microparticles interact with leukocytes and other inflammatory cells and can amplify inflammation in the arterial wall [62].

Besides contributing to the initiation and progression of atherosclerotic lesions, platelets trigger the acute onset of arterial thrombosis when these lesions rupture or undergo erosion [60]. The exposure of the thrombogenic substrates to circulating platelets challenges platelet recruitment to the injured vessel wall in a well-coordinated series of events: platelet ‘arrest’ onto the exposed subendothelium; recruitment and activation of additional platelets through the local release of major platelet agonists; and stabilization of the platelet aggregates [64]. These events ultimately result in the formation of a non-occlusive or occlusive platelet-fibrin thrombus. Acute occlusive coronary thrombus growth is most frequently the cause of acute coronary syndromes (ACS) and in some cases even of sudden coronary death [65].

#### 1.3.2. Platelets in Diabetes Mellitus

Diabetes mellitus is a multifactorial disease closely associated with both micro- and macrovascular complications and a high risk of atherothrombotic events. Platelets play a major role in the pathophysiology of DM. Platelets of DM patients are indeed characterized by dysregulation of several signaling pathways leading to platelet activation, which represents an early event in the natural history of DM [66,67]. The detrimental metabolic state that precedes and accompanies diabetes, characterized by acute hyperglycemia, glycemic variability, and insulin resistance, is thought to be responsible for the alterations in platelet function seen in DM. These metabolic abnormalities may affect platelet transcriptome and/or posttranscriptional regulation through intermediate mediators, such as oxidative stress with isoprostane formation, inflammatory molecule production, endothelial dysfunction with circulating endothelial cells and microparticles release, and cross-talk between cells with miRNA exchange through circulating microparticles [68]. The net result of these influences is platelet hyperreactivity, as reflected by enhanced platelet TF expression and expression of TF-positive platelet-leukocyte aggregates; increased expression of adhesion molecules such as P-selectin; and increased arachidonic acid metabolism and enhanced TXA2 biosynthesis [68]. In turn, hyperactivated platelets have fundamental roles in both the development and the propagation of sustained inflammation in DM, are increasingly recognized as the culprit cells implicated in the propensity to atherothrombosis in the setting DM, and contribute to diabetes vascular complications. Thus, platelets appear as both targets and effectors in the pathophysiology of DM, carrying and transducing metabolic derangement into vascular injury [67].

Future efforts to decrease the thrombotic burden in diabetes should target specific disease-based mechanisms. In this perspective, high-throughput techniques are fundamental since they offer a unique opportunity to deciphering the molecular networks altered by the metabolic derangement associated with type 2 diabetes mellitus (T2DM), such as the platelet transcriptome and proteome composition and/or post-transcriptional regulation [68].

#### 1.3.3. Platelets in Cancer 

Tumorigenesis is a multistep process requiring concerted changes in both tumor cells and the tumor microenvironment. Experimental evidence has highlighted platelets as active players in all steps of tumorigenesis including tumor growth, tumor cell extravasation, and metastasis [69,70]. They infiltrate into the tumor microenvironment to directly interact with cancer cells and can activate the same proliferative pathways that are activated through oncogenic mutations, thus contributing to the initiation and progression of disease. Platelets also exert anti-apoptotic roles in both hematopoietic and solid tumor malignancies, thus sustaining tumor cell survival [69,70]. Moreover, platelets have the ability to deliver multiple proangiogenic factors to the tumor, including vascular endothelial growth factor, platelet-derived growth factor, fibroblast growth factors, and MMPs, as well as the ability to stimulate the expression of proangiogenic factors by the tumor cells. In that way, platelets promote the neovascularization needed to assure an adequate blood supply for delivering necessary nutrients, removing waste, and oxygenating the tumor [69]. In the circulation, platelets help circulating tumor cells to escape the deadly attack of the immune system by building a partial physical barrier toward natural killer cells, through the formation of platelet-tumor cell aggregates, and by interfering with the recognition of cancer cells by natural killer cells, through the transfer of “normal” major histocompatibility complex class I molecules onto the surface of tumor cells [69,70]. This ability of platelets to protect tumor cells in circulation from immune surveillance is likely to significantly contribute to the metastatic process. Additionally, by activating the platelet-derived transforming growth factor-β/Smad and nuclear factor kB pathways, platelet-derived transforming growth factor-β facilitates an invasive epithelial-to-mesenchymal transition phenotype in cancer cells and increase metastases [70]. In addition, platelets may contribute to metastasis, helping circulating tumor cells to attach to the endothelium, providing signals to establish a pre-metastatic niche, and facilitating extravasation at a distant site [69,70,71]. Finally, platelets even influence the sensitivity of chemotherapy and other targeted therapies in cancer patients [70]. On the other hand, tumor cells mediate platelet activation, leading to platelet aggregation and the release of platelet-derived growth and proangiogenic factors in the tumor microenvironment, which may contribute to tumor growth and angiogenesis and further magnifies the pro-coagulant milieu generated by the interaction between platelets and cancer cells [70]. 

Overall, increasing evidence supports the notion that in a cancerous setting, the normal hemostatic role for platelets can be hijacked to promote tumor growth, survival and metastasis, and that cancer cells and platelets maintain a complex, bidirectional communication. Based on this evidence, it is to be hoped that developing methods to specifically target platelet interaction with tumor cells without interfering with normal platelet functions could provide a significant advance in the treatment of cancer patients, especially in the metastatic setting [69,70,72].

#### 1.3.4. Platelets in Lung Diseases

Platelets and the lungs have an intimate relationship: the lungs are reservoirs for megakaryocytes, the precursor cell in thrombopoiesis, and platelets sustain hemostatic and inflammatory defense of the healthy lung. However, experimental and clinical evidences indicate that platelets are also effectors of injury in a variety of pulmonary disorders and syndromes [73]. Alterations in platelet numbers and function occur in primary lung infections, such as influenza, and in systemic infectious syndromes that involve the lungs and pleurae, such as bacterial sepsis [73]. In addition, platelets are implicated in chronic and intermittent inflammatory lung syndromes, including chronic obstructive pulmonary disease, cystic fibrosis, and asthma [73]. Moreover, activated platelets contribute to initiation and/or amplification of alveolar damage in the acute respiratory distress syndrome (ARDS) and alterations in platelet number and function influence the natural history of this syndrome [73]. Additionally, platelets contribute to pathological lymphangiogenesis in the lung, pulmonary embolism, and pulmonary hypertensive disorders [73,74]. Additionally, platelets are determinants of lung metastasis in experimental and clinical neoplasia [75]. Based on this evidence, it has been highlighted that changes in the transcriptome, proteome, and metabolome of platelets and their megakaryocytic precursors in lung diseases need to be examined to better understand the protean activities of platelets in pulmonary pathophysiology [75].

#### 1.3.5. Platelets in Neurological Disorders

The findings that platelets have many biochemical similarities with neurons, as it is the storage and release of neurotransmitters from platelets such as serotonin, glutamate and dopamine and the expression of neuron-related proteins such as N-methyl-D-aspartate receptors, make them an interest contributor in neurodegenerative diseases (reviewed in [76]). 

Platelets show high expression of several proteins associated with the development of Alzheimer’s disease (AD), such as the amyloid precursor protein [77] and tau protein [78]. In addition, platelets are the main source of 5-hydroxytryptamine and contain all the enzymatic machinery to amyloid precursor protein processing [79], and they contain concentrations of its isoforms equivalent to those found in brain [80]. Thus, platelets represent an important peripheral source of amyloid precursor protein [81] and its release from activated platelets contributes to vascular amyloid deposits. Platelet dysfunction and amyloid precursor protein processing abnormalities are believed to occur rather early during the course of AD [82].

Further, platelets adhering to the vascular wall, lead to sustained platelet recruitment and potentially to full vessel occlusion, thus producing increased Aβ40 peptide secretion, development of cerebral amyloid angiopathy, dementia and, finally acceleration of the progression of AD [83].

Activated platelets might be crucial in the development of other diseases, like Parkinson’s, multiple sclerosis and others. Their potential role in these diseases is the topic of a recent review [76] and is not further described herein because up to now proteomics has been applied only in AD. Platelets could somehow reflect what is happening in the central nervous system along the course of neurodegenerative pathological states, and therefore could be promising biomarkers for early onset diagnosis of a pathological condition.

### 1.4. Platelet Proteins as Targets for Antiplatelet Therapy

Antiplatelet therapy is widely used for the treatment and prevention of patients with thrombotic cerebrovascular or cardiovascular diseases, but also patients who have undergone angioplasty, stent procedures or coronary artery bypass surgery [84]. Antiplatelet therapy with one or more drugs decreases platelet aggregation and activation.

Currently, the principal platelet proteins used as targets in antiplatelet therapy are cyclooxygenase-1 (COX-1), P2Y_12_ receptor and α_IIb_β_3_ integrin [18].

Cyclooxygenase-1 regulates the production of prostaglandins, which control platelet aggregation and activation, and it is inhibited by irreversible cyclooxygenase inhibitors that show anti-inflammatory effects [85]. In the 1960s, aspirin (ASA) was the first valid antiplatelet drug, and now it still used to prevent cardiovascular complications. ASA has an inhibitory effect by irreversible acetylation of COX-1, thus blocking the prostaglandin and TXA2 synthesis [86].

The P2Y_12_ receptor is antagonized by thienopyridines (e.g., clopidogrel and prasugrel), which block the ADP binding to this receptor, inhibiting platelet activation and Gi protein association [23]. Initially discovered to reduce the occlusion of coronary stents, today these drugs have a wider application in the prevention of cardiovascular events in high-risk patients, mostly in combination with ASA. The combination of an ADP inhibitor and ASA is known as dual antiplatelet therapy [87].

The α_IIb_β_3_ integrin is a platelet receptor for fibrinogen, fibronectin, vitronectin, and von Willebrand factor (vWF). It is targeted by the GPIIb/IIIa inhibitors (e.g., abciximab), which are primarily used to treat acute coronary syndromes (ACS) and thrombotic complications.

PAR-1 antagonists are a recently discovered class of antiplatelet drugs, among which vorapaxar selectively inhibits thrombin-induced platelet aggregation and it is used for the treatment of patients who have suffered a myocardial infarction (heart attack) or peripheral arterial disease [88]. Vorapaxar has a limitation due to its long half-life and slow off-rate, thus it is contraindicated in patients with a history of stroke or transient ischemic attack [89]. In the last few years, the search for alternative PAR-1 antagonists with better pharmacokinetic profiles has been triggered (e.g., PZ-128).

The study of new drugs and their platelet targets is constantly growing, and some promising targets are still under clinical investigation to discover novel potential biomarkers for accurate therapeutic approaches [86,89]. Moreover, the choice of more selective platelet antagonists, as well as the monitoring of therapeutic antiplatelet regimens, turns out to be crucial to keep the side effects to a minimum. It is well known that the response to antiplatelet therapy varies from one patient to the next, and unfortunately, several side effects have been reported [86]. Although the antiplatelet therapy is able to prevent death and complications in patients with high risk of CVDs, the problem of controlling bleeding and the other side effects persists by pointing out the importance of personalizing the drug therapy and improving approaches for monitoring [90]. The search for better and safer antiplatelet drugs is one of the principal investigation directions in basic and clinical research.

## 2. Overview of Proteomics of Platelets

### 2.1. Role of Proteomics in Platelet Study

Because of the important role of platelets in the pathophysiology of several disease conditions, intense research has been done during the last decades aimed at identifying activation biomarkers and potential therapeutic targets for several diseases. Indeed, several antiplatelet therapies have been successfully discovered and applied in clinics, although core issues remain regarding the highly variable pharmacological response of patients. Therefore, novel approaches based on “omic” technologies have been developed to overcome these limitations. Indeed, the field of “omics” has a central role in the monitoring of patients for development of the so-called precision medicine, which is a relatively new concept based on the identification of the optimal prevention and treatment approaches that are effective for each patient considering individual differences in genetics, lifestyle, environmental and health factors. Nowadays, proteomics has become a crucial technology to better understand the cellular and molecular mechanisms as well as individual protein profiling, which could be used to individualize the patient care and develop new therapeutic strategies. Proteomics has significantly helped to describe the principal signaling pathways in platelets, leading to the characterization of new signaling proteins and receptors. Platelets are a rich source of biomarkers, containing several bioactive proteins in the granules, which can be secreted upon platelet activation, as well as expressing many receptors that can contribute to disease progression. Thus, platelet-derived biomarkers have attracted great interest in biomedical research in order to apply them in monitoring disease course and treatment response. This review summarizes the application of proteomics in this field of research aiming to discuss the latest advances in platelet biomarker research (Table 1).

### 2.2. Proteomic Characterization of Platelets

Several key issues should be taken into account for proteomic studies to prevent the activation or degradation of platelets during the collection and preparation, thereby causing morphological changes that lead to the release of granular content and alterations in protein expression. Platelet activation can be prevented by using a pharmacological pre-treatment of sample or by an accurate restriction to the use of activating surfaces and conditions [162]. According to the proteomic study goals, it is very important the choice of an appropriate pharmacological agent considering its mechanism of action that could interfere with the pathways of interest or alter the protein profile. Moreover, it is recommended to avoid glass and polystyrene plastic surfaces, changes in temperature or pH, strong mechanical forces and long static incubations. It is advisable to handle and store platelets in polypropylene, polyethylene or polycarbonate tubes.

Platelets can be obtained from relatively small volumes of blood draw, and only a few micrograms of platelet proteins are necessary for a proper proteomic analysis. The blood draw occurs in anticoagulated vacutainer tubes, the use of syringes or long tubing is not recommended, while large bore needle is preferred. It is also desirable that blood and platelets handling is at room temperature or 37 °C and the isolation of platelets occurs immediately after the blood collection to avoid their activation and proteomic profile modifications. 

Moreover, in proteomic experiments on healthy platelets, it is crucial that healthy donors are not under medication for at least 10/15 days at the time of collection [163]. 

**Figure 3:**
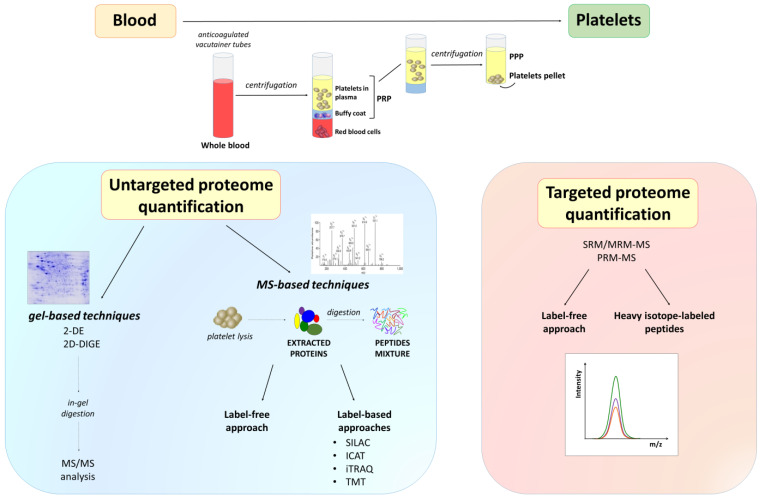
Proteomic characterization of platelets. Overview of the typical proteomic workflow used for platelet research. Platelets are obtained by purifying PRP from blood cells and subsequent centrifugation, and then lyzed to obtain proteins that are enzymatically digested. Platelet global proteome (all peptides) or subproteomes and PTMs (after an enrichment step) can be analyzed using MS. Both gel-based and gel-free techniques have been developed to study platelet protein composition and interactions. MS-based quantification can be performed by label-free techniques or using chemically (iTRAQ, ICAT, TMT)/metabolically (SILAC) introduced stable isotope labels. Finally, candidates are chosen for a targeted absolute quantification, which is usually performed by SRM/MRM or PRM. 2-DE, two-dimensional gel electrophoresis; 2D-DIGE, 2-dimensional difference gel electrophoresis; ICAT, isotope-coded affinity tag; iTRAQ, isobaric tags for relative and absolute quantification; MRM, multiple reaction monitoring; PPP, platelet-poor plasma; PRM, parallel reaction monitoring; PRP, platelet-rich plasma; PTMs, post-translational modifications; SILAC, stable isotope labeling by amino acids in cell culture; SRM, single reaction monitoring; TMT, tandem mass tags.

The choice of method for platelet isolation is essential for highly pure platelet preparation, reducing contamination and activation. The contamination from other blood cells, such as red cells and leukocytes, should be avoided, preferring the upper third of the platelet-rich plasma (PRP) and, eventually, the use of white cells-reduction filters (Figure 3). To prevent platelet activation during centrifugations, apyrase or prostaglandin E1 can be added to anticoagulated whole blood or PRP. After the centrifugation of the whole blood, three distinct layers can be formed: the bottom layer of red blood cells, the middle layer or “buffy coat” of white blood cells, and the top layer of PRP [164]. Platelets resuspended from PRP should be centrifuged again to pellet residual contaminating erythrocytes and leukocytes and washed in a suitable buffer [165]. It was reported in literature that a platelet pellet obtained from 1 mL of PRP can deliver about 1–1.5 mg of total protein [162].

Several PRP preparation protocols are described in the literature, and it was observed that platelet concentration is closely correlated with both the collected blood volume and use of a centrifugal force device [166]. Thus, in future, it will be crucial to standardize the procedure for high-quality proteomic studies on platelets.

Platelet protein abundances have an extremely large dynamic range, making their analysis in plasma very challenging. Therefore, the improvement and standardization of the protein extraction procedure is a priority task. Protein extraction includes the use of sample buffers where platelet proteins are solubilized prior to their analysis in MS. Both gel-based and gel-free techniques have been developed to study platelet protein composition and interactions in order to characterize the processes in which platelets are involved in healthy and disease conditions (Figure 3). Gel-based methods have several limitations in their application, but allow a greater resolution of protein isoforms [167]. Two-dimensional gel electrophoresis (2-DE) is a powerful gel-based technology that has been extensively used for the analysis of platelets.

Thanks to increasingly powerful mass spectrometers and more accurate quantitative methods, the gel-free proteomic techniques allowed for the limitations of gel-based methods to be overcome. Gel-free methods usually require less sample material and provide higher resolution, sensitivity and reproducibility, as well as being easily automated and less time-consuming.

A typical proteomic workflow used for platelet research involves multiple steps [18] (Figure 3). Platelets are isolated from human blood samples and lyzed to obtain proteins, that are then enzymatically or chemically digested, providing complex peptide mixtures available for both a global proteome (all peptides) and a PTMs proteome analysis using MS. LC is usually applied before the MS analysis as a prefractionation method, and in case of a PTMs proteome investigation, a prior purification step allows enrichment of post-translationally modified peptides. These discovery approaches return an extensive list of proteins that can be used to select candidates for further absolute quantification, which is usually performed using single reaction monitoring/multiple reaction monitoring (SRM/MRM) or parallel reaction monitoring (PRM). Finally, validation studies are conducted aimed to confirm potential biomarkers that can be used in diagnostics and therapeutics. 

Platelet sample MS-based quantification can be performed by label-free techniques or using stable isotope labels (Figure 3). Label-free approaches are applied for the relative quantification of peptides, in which direct comparisons of MS intensities or tandem mass spectrometry (MS/MS) spectra are performed between different samples analyzed separately. Instead, labelling can be chemically performed, such as using isobaric tags for relative and absolute quantification (iTRAQ) [152], isotope-coded affinity tagging (ICAT) [168] or tandem mass tags (TMT) [169], or metabolically using stable isotope labelling by amino acids in cell culture (SILAC) [170]. In labelling strategies, a specific mass is added to each peptide of interest, and it is possible to mix differentially labelled samples using diverse stable isotopes and discriminate them by mass shifts in MS and MS/MS spectra.

## 3. Detailed Review of the Literature of Proteomics of Platelets

### 3.1. Global Proteome of Platelets

The global investigation of the platelet proteome is a very important step to understand the multifunctional nature of platelets and discover potential biomarkers involved in the progression of CVDs and cancers [171]. Numerous platelet proteomic studies have been performed during the last 20 years and many proteins have been identified in human platelets, which include cytoskeletal and signaling proteins, cell surface receptors, and proteins of the proteasome and immunoproteasome subunits [124].

In global proteome studies, platelet proteins can be enriched by precipitation with trichloroacetic acid/acetone or with an ethanol/dialysis method or other commercial precipitation kits [19]. Moreover, platelet membrane disruption can be done by lyzing cells in liquid nitrogen or using detergent hypotonic buffers. 

#### 3.1.1. Global Proteome of Platelets in Healthy and Disease Conditions

Initially, the attention was focused on studying the proteome of platelets in their basal state. Marcus et al. performed the first analysis of human platelet proteome using MS, allowing the identification of 186 proteins using a matrix-assisted laser desorption/ionization-time of flight MS (MALDI-TOF MS) technique [91]. The number of identified human platelet proteins was then extended to 760 features using narrow pI range 2-DE followed by high-throughput MS/MS [96].

Gel-free proteomic technologies and MS analysis have provided a more complete characterization of the platelet proteome in the following years. A non-gel proteomic technology known as combined fractional diagonal chromatography (COFRADIC) has been applied to isolate N-terminal peptides, which were then analyzed by nanoLC-MS/MS, and 264 proteins and 78 in vivo-acetylated proteins were identified in cytosolic and membrane skeleton fractions of human platelets [95]. The same group specifically isolated cysteine-containing peptides using the same technique and identified their precursor proteins by nanoLC-MS/MS analysis [97]. These proteins are not usually identified by 2-DE, being of low abundance and too hydrophobic. 

Burkhart et al. described the first comprehensive study of protein networks and pathways in human platelets from healthy donors using a quantitative proteomic approach in a two-pronged way (normalized spectral abundance factor and iTRAQ labeling) and nanoLC-MS/MS analysis [124]. The author identified almost 4,000 unique proteins and more than 2.500 phosphorylation sites, evaluating the intersubject and intrasubject variance. This study has been a starting point for further quantitative proteomic analysis, in which differences in a specific disease condition or in response to antiplatelet treatment have been evaluated.

MS-based proteomic analysis allowed the assessment of the differences in platelets with different origin, for example, derived from umbilical cord blood and adult peripheral blood [148]. 

It is well known that changes in proteins might reflect a pathological profile, allowing the early recognition and monitoring of a specific pathological condition, as well as the discovery of potential drug targets [172]. One of the first platelet proteomic studies in clinical research was performed by Arias-Salgado et al. on the role of platelets in arterial thrombosis [108]. Platelet protein content from patients who had suffered an arterial thrombosis episode was compared with healthy subjects by 2-DE and MALDI-TOF MS analysis. The observed differences in the protein profiles were evident months after the acute thrombotic event and reached control levels only after years, confirming persistent platelet hyperactivity after the thrombotic event. 

Platelets have an important role in the development and progression of atherosclerotic plaque, but it is not clear if coronary artery disease (CAD) promotes specific changes in the human platelet proteome. Banfi et al. applied 2-DE and nanoLC-MS/MS to investigate protein patterns of resting platelets from patients with stable or acute CAD and subjects without CAD for the first time [116]. The identified protein changes have never been previously connected with this pathological condition, and they indicated a platelet activation.

Several studies have also applied platelet proteomics in ACS patients to identify new valid disease biomarkers. Proteomes of circulating platelets from patients with non-ST segment elevation acute coronary syndrome (NSTE-ACS) were investigated in comparison with stable coronary artery disease (SCAD) controls by 2-DE and MS [117]. Differentially regulated platelet proteins were associated with increased platelet activation in NSTE-ACS patients in parallel to the acute event. Moreover, the authors demonstrated that proteomics could be used to follow platelet proteome changes during disease follow-up. Indeed, they compared the platelet proteome in patients with ST-elevation myocardial infarction (STEMI) and matched SCAD controls over time, identifying the main pathways involved and highlighting an increased activation of the sarcoma protooncogene tyrosine-protein kinase pathway and collagen receptor GPVI signaling cascade in STEMI patients [121]. However, a platelet proteomic study by López-Farré et al. produced conflicting results, showing a downregulated expression of proteins involved in cellular cytoskeleton, glycolytic pathway and cellular-related antioxidant system in ACS patients [122].

Some years later, Vélez et al. compared the proteome of intracoronary and peripheral arterial platelets from STEMI patients by two-dimensional difference gel electrophoresis (2D-DIGE) and MS, demonstrating that the upregulation of specific proteins in intracoronary platelets was probably due to PTMs and protein synthesis following acute platelet activation and inflammation at the culprit site [142].

Platelets also play an important role in tumour growth and their characteristics are affected by cancer presence. Recently, Sabrkhany et al. performed the first study to measure the effect of cancer on platelet proteome of patients with early-stage lung or head of pancreas cancer [154]. Using nanoLC-MS/MS analysis, a total of 4.384 unique platelet proteins was identified, among which 85 were significantly modified in early-stage cancer compared to controls. Moreover, a tumour type-dependency of platelet changes was reported, probably due to differences in cancer cell secretome and/or tumour localization. Tumour resection led to an additional proteome change, and the levels of 81 differently expressed platelet proteins normalized after tumour resection. This pioneering study proves that platelets can be a promising source of candidate biomarkers of early-stage cancer and it would be interesting to monitor platelet response to different types of cancer treatment.

In addition, platelets are considered a peripheral model to study the pathogenesis of AD. Platelet protein expression was investigated using MS-based proteomics in patients with mild cognitive impairment (MCI), AD, and healthy subjects, and significant differences were described in proteins involved in cytoskeletal regulation, inflammation and immune response [155]. Moreover, platelet amyloid precursor protein and amyloid-β peptides were increased in patients. Some changes occurred at the early stages of the disease, thus representing potential new pathways involved in AD pathogenesis.

#### 3.1.2. Global Proteome of Activated Platelets

Proteomic techniques have been also applied to study the proteome of human activated platelets and thus clarify the mechanisms at the basis of activation and aggregation. The most widely studied pathway has been thrombin-receptor signaling. Gevaert et al. applied a combination of 2-DE and MALDI-TOF MS to study cytoskeletal preparations of human basal and thrombin-stimulated platelets, confirming the translocation of several proteins to the actin cytoskeleton of platelets upon platelet-thrombin activation [92]. Later, Maguire et al. proposed an immunoprecipitation-based method for the isolation of the phosphotyrosine proteome from both resting and thrombin-activated human platelets, and a great number of tyrosine phosphorylated signaling proteins has been uniquely detected in activated platelets using 2-DE and MALDI-TOF MS [93].

García et al. also performed a differential proteome analysis of intracellular signaling cascades using 2-DE coupled with nanoLC-MS/MS in human platelets stimulated with thrombin-receptor activated peptide (TRAP), which is a synthetic peptide with full agonist properties for thrombin receptor PAR-1 activation [98]. Moreover, the same group investigated the proteome of collagen-related peptide (CRP)-stimulated platelets to obtain information on the intracellular signaling events that take place upon activation of GPVI for the first time [102].

Recently, a label-free MS quantitative proteomic analysis of β-catenin immunoprecipitates was carried out from human platelets under resting and TRAP-activation, showing a strong association of β-catenin with cadherin junction proteins and regulators of WNT signaling following platelet activation [156]. 

Platelet stimulation with other agonists has been also studied. For example, a comprehensive molecular investigation of ADP-induced protein phosphorylation has been performed to understand platelet changes during aggregation and secretion. Temporal phosphorylation patterns in human platelets have been studied by quantitative MS after stimulation with ADP and ADP + Iloprost [149], providing a list of more than 4,000 phosphopeptides and an inverse regulation for a set of phosphorylation sites by inhibition with Iloprost.

In another study, the activation state of GPVI signaling in STEMI patients has been evaluated by combining a tyrosine phosphoproteomics-based approach and immunoblotting-based validation assays [143]. Platelets from STEMI patients and SCAD controls were activated with CRP, and a panel of GPVI signaling biomarkers was identified to be hyperphosphorylated in STEMI. Their validation was conducted on systemic and intracoronary blood from an independent larger cohort of patients, demonstrating that STEMI patients were characterized by an altered activation state of GPVI signaling, which could be a promising antithrombotic target for myocardial infarction.

Moreover, a targeted quantitative proteomic approach using LC-MRM MS has been applied to study small GTPase isoforms at different time points from human platelets in response to several agonists [138]. In particular, the active small GTPases were precipitated using a specific resin, separated by Sodium Dodecyl Sulphate-PolyAcrylamide Gel Electrophoresis (SDS-PAGE), and tryptic digested with the addition of heavy isotope labelled peptides for the LC-MRM MS analysis. This study showed a time-resolved coactivation of multiple small GTPase isoforms in response to agonists and their differential activation in response to inhibitor treatment.

### 3.2. Platelet Subproteomes

Besides global proteomic studies, it is essential to study platelet subproteomes (i.e., microparticles, specific granule releasate and membrane) to characterize relevant proteins that are expressed in specific compartments and releasates, leading to a better understanding of their localization and function in healthy or disease conditions.

The platelet releasate includes all inflammatory and vasoactive biomolecules that are secreted by activated platelets, in granules or microvesicles [19]. Strong agonists, such as thrombin, PAR-1 and 4, collagen and collagen-related agonists, can be used to stimulate platelets and induce a rapid release of cargos [173]. Instead, other specific agonists, like ADP or TXA2, stimulate a slower granule secretion response with differential protein release. Thus, it is possible to regulate platelet release through different agonist concentrations and combinations, as well as incubation times and temperatures. After platelet activation, the supernatant can be further ultracentrifugated to obtain the soluble releasate proteome on one hand and PMP proteome from the pellet on the other. 

The first proteomic analysis of the platelet releasate, a fraction highly enriched for granular and exosomal contents, demonstrated the localization of platelet proteins in human atherosclerotic lesions [99]. After platelet stimulation with thrombin, 2-DE and MS allowed the identification of more than 300 proteins, among which three of them showed a potential relevance in the pathogenesis of atherosclerosis because of their presence in atherosclerotic lesions and absence in normal vasculature.

In another study, TRAP-induced releasate from platelets of healthy volunteers was analysed using high resolution and high mass accuracy hybrid ion-trap Fourier-transform mass spectrometry in a GeLC-MS/MS workflow [111], providing an extensive list of proteins that included both soluble proteins released from granules and proteins present in microparticles.

A releasate proteomics approach using 2D-nanoLC-MS/MS was also applied by Wijten et al. to quantitatively determine proteins released from platelets of healthy donors activated with a combination of thrombin and collagen [128]. The authors quantitatively monitored the concentration changes of about 4500 proteins, and after stimulation, some of them were significantly released spanning a concentration range of ≥5 orders. A comparison between the platelet releasates following the activation with thrombin and collagen was also performed using 2D-DIGE and nanoLC-MS/MS [139], demonstrating that more than 100 protein spots significantly varied between the two conditions, and several identified differences corresponded to PTMs, mainly proteolysis induced by thrombin. These results, although obtained in studies with small sample sizes, confirmed that the platelet secretome varies depending on the stimulus.

Parsons et al. performed a study on a greater cohort of healthy donors using a label-free quantitative proteomic approach to evaluate thrombin-induced platelet releasates [157], and they quantified 277 proteins, most of which coincided with the protein composition of platelet-derived exosomes. Since these proteins were both soluble and vesicle-derived and the population variance was very low, this study could represent a useful platform for diagnostic profiling of platelet-related diseases.

The proteomic studies of platelet releasates upon stimulation with other agonists remain scarce. MS-based quantitative proteomics has been applied to study healthy platelet releasates upon stimulation with PAR-1 and PAR-4 agonists [131], and there was no evidence of a differential α-granule release in these conditions.

As mentioned above, platelet–collagen interactions are key events in the pathophysiology of CVDs and platelet collagen receptors, especially GPVI, are involved in haemostasis and arterial thrombosis, representing highly interesting potential targets for antiplatelet drugs. Human platelet proteome has been investigated by 2D-DIGE and MS after stimulation with an activating monoclonal antibody specific for GPVI [118], and GPVI activation induced differential changes in abundance of releasate proteins, in particular a significant increase of ERp57, which regulates platelet aggregation and platelet-dependent coagulation.

#### 3.2.1. Platelet-Derived Microparticles (PMP)

Platelet-Derived Microparticles are very small vesicles, ranging from 100 nm to 1.0 μm, released from blood activated platelets and represented the main population of circulating microparticles in healthy subjects [174]. Several stimuli can promote the PMPs release from the platelet plasma membrane or directly from the megakaryocytes. PMPs generation can be mediated by two conditions: (1) reorganization of the actin cytoskeleton and formation of platelet vesicles mediated by GPIIb/IIIa receptors; (2) high cytosolic calcium concentration that leads to the activation of calcium protease and protein kinase C, and, consequently, PMPs formation [45].

Platelet-Derived Microparticles carry platelet-derived cytokines, enzymes, nucleic acids, lipids, and also transcription factors, that are involved in inflammation, angiogenesis, blood coagulation, immune response, and intercellular communication [175]. In healthy conditions, human platelets regularly release PMPs with multiple physiological effects. PMPs play an important role in haemostasis, but their increase in the blood concentration is reported in several pathological conditions, such as tumour progression, cardiovascular and cerebrovascular diseases [176,177,178]. For these reasons, PMPs are extensively studied because they can be useful biomarkers for the detection of patients in specific pathological states or potential drug targets.

For a correct PMPs estimation, it is important to process samples immediately after blood draw, because PMPs concentration increases in plasma with time [179]. Platelet-poor plasma (PPP) contains PMPs and microparticles from other cells, so it is necessary to isolate PMPs using specific surface protein labelling with fluorescent tagged antibodies. Ultracentrifugation is also required to separate exosomes from PMPs, while ultrafiltration with size exclusion chromatography and affinity chromatography can be used for the isolation of extracellular vesicles. PMPs levels are usually measured by microscopy, enzyme-linked immunosorbent assays and flow cytometry.

Recently, Ponomareva et al. performed a study on the structural heterogeneity of PMPs, in both resting and activated platelets, and proposed a morphological classification of PMPs based on their structure, size, mechanisms of formation, and the presence of inclusions/organelles inside them, for a better understanding of their specific physiological and pathological effects [174]. 

Several proteomic approaches can be applied for protein profiling of PMPs, such as SDS-PAGE, 2-DE, Western blotting and MS combined with chromatographic techniques. PMPs contain a unique subset of proteins, and they can transfer receptors, stimulate the release of cytokines, increase expression of adhesion molecules and promote intracellular signaling. The first proteomic analysis of microparticles from activated platelets was carried out using 1D SDS-PAGE and nanoLC-MS/MS and more than 500 proteins were identified, among which were membrane surface proteins and chemokines [100]. Later, a proteomic analysis on human platelets activated with thrombin and collagen allowed PMPs to be distinguished into different size classes based on protein components using gel filtration chromatography and 2D-nanoLC-MS/MS [113]. The study showed that large PMPs derived from the platelet plasma membrane, while smaller particles from internal storage vesicles. Moreover, the authors observed a decrease of mitochondrial proteins with reducing fraction size, an enrichment of plasma membrane and cytoskeleton-associated proteins in the smaller size fractions, and the absence of α-granule proteins in the larger fractions, thus confirming that smaller PMPs called exosomes originate from α-granules. 

A shotgun proteomic approach by nanoLC-MS/MS and fractionation with hydrogel nanoparticles were applied to study proteomic profile of ADP-induced PMPs allowing the identification of about 600 proteins, among which the majority was distributed in the subsection cytoplasm and more than half had binding and catalytic activity [129]. Instead, Kasprzyk et al. evaluated the protein composition of platelet microvesicles (PMVs) from thrombin-activated platelets of healthy donors using a nanoLC-MALDI-TOF/TOF MS analysis, and more than 400 proteins were identified, among which 123 were PMV-specific [151]. 

Several proteomic studies also focused the attention on the PMPs characterization after platelet activation with different agonists. For example, several proteins were found to be differently expressed between PMPs derived from platelet activation with thrombin and shear stress, thus demonstrating differences in PMPs amount and proteome depending on the activation stimulus [125]. Milioli et al. presented the most detailed quantitative proteomic analysis of PMPs from differentially activated platelets of healthy volunteers using iTRAQ labelling and reversed-phase nanoLC-ESI-MS/MS [140]. More than 3000 proteins were identified, and several membrane and soluble proteins were significantly different in at least one of the analyzed conditions, confirming that PMP proteome depends on the type of agonist involved in platelet stimulation.

Besides microparticles, platelets also release exosomes from the exocytosis of multivesicular bodies and α-granules, ranging from 30 to 100 nm in diameter. Aatonen et al. proposed an isolation method for platelet-derived extracellular vesicles (EVs), including both microparticles and exosomes, which in combination with SDS-PAGE and nanoLC-MS/MS allowed to show high EVs heterogeneity between the vesicle subpopulations and after the different activations [136]. Moreover, this study revealed higher protein content in exosomes of unstimulated platelets, suggesting the important role of exosomes also in the basal vesicle release.

PMPs have great importance in several diseases and increased circulating levels have been observed in patients. For example, higher levels of circulating PMPs were found in acute myocardial infarction [180] and it was assumed that their proteome could be a relevant source of biomarkers for the disease. Moreover, it was noted that PMPs are important mediators between platelet activation and oxidative stress, which has a key role in the atherothrombosis [181].

#### 3.2.2. Platelet Granule Releasate

As mentioned before, platelets contain three types of specific granules, whose proteome has been investigated by several groups. Hernandez-Ruiz et al. isolated human highly enriched platelet dense granule fractions by density gradient isolation method for the first time, and analyzed their soluble protein composition by 2DE coupled to MALDI-TOF MS and nanoLC-MS/MS [105]. In particular, the expression of 14-3-3ζ was studied in sections of abdominal aorta of patients with aneurysm and higher levels were found in the extreme luminal side of the atherosclerotic plaque. 14-3-3ζ is secreted after platelet activation, regulates platelet signaling and GPIb-IX-V complex, and it has a relevant role in the atherosclerosis progression.

In the same year, for the first time, Maynard et al. extensively characterized the human platelet α-granule proteome from healthy donors using a sucrose gradient ultracentrifugation for the isolation of a subcellular fraction enriched in α-granules, and SDS-PAGE combined with nanoLC-MS/MS for the identification of almost 300 proteins [106]. Later, the same authors reported that soluble and membrane-bound α-granule proteins were markedly reduced or undetected in a Gray Platelet Syndrome patient, supporting the presence of the so-called “ghost granules” in platelets of patients and pointing out the failure of megakaryocytes to package secretory proteins into α-granules [119]. 

In another study, a comprehensive characterization of the platelet granule proteome was performed by MS and 827 proteins were identified, some of them belonged to the major histocompatibility complex class I antigen presentation pathway [137]. These proteins were present in α-granules and located on the plasma membrane upon platelet activation, suggesting a potential role of the granules in platelet-related immune modulation. 

#### 3.2.3. Platelet Membrane Proteins

Platelet membrane proteome is a highly platelet-specific subproteome and easily accessible, thus representing a good candidate for disease biomarkers and drug targets. Membrane proteins are not resolved properly in 2-DE analysis, mainly due to their poor solubilization, thus a combination of chaotropes and detergents is usually applied to improve their solubility in the first dimension or a sample prefractionation to obtain membrane preparations before the SDS-PAGE analysis and nanoLC-MS/MS. The enrichment of membrane subproteome prior to nanoLC-MS/MS reduces the number of cytoskeletal proteins and promotes the detection of less abundant cell-surface transmembrane proteins. 

One of the first studies focused on human platelet membrane proteome was performed by Moebius et al. using SDS-PAGE and nanoLC-MS/MS analysis with an identification of nearly 300 proteins [101]. The most comprehensive study of platelet membrane proteome resulted in the identification of 1.282 proteins using three complementary strategies: 1D SDS-PAGE followed by nanoLC-MS/MS, strong cation exchange (SCX) and reverse-phase chromatography before MS, and COFRADIC. Almost 70% of the identified proteins were of membrane origin, both plasma membrane and other membrane-bound compartments [114].

Platelet membrane proteins may serve as biomarkers or new therapeutic targets in several pathological conditions and some studies have focused their attention in this direction. Donovan et al. investigated the potential of the platelet membrane-associated protein changes in the differentiation of patients with probable AD from non-cognitive impaired controls [130]. Significantly changed protein abundances were determined by SDS-PAGE and nanoLC-MS/MS in patients, confirming the enhanced platelet activation in circulation. This study demonstrated that cell-surface platelet membrane proteins could be useful blood-based biomarkers and potential targets for diagnostic screening approaches. In another study, human platelet and mouse megakaryocyte membrane proteins were identified using a proteomic approach with three different enrichment techniques before nanoLC-MS/MS [107]. Several surface transmembrane proteins were identified, including the immunoglobulin superfamily member G6b. In particular, the expression of G6b-B isoform was investigated using specific antibodies and it was found that this protein undergoes tyrosine phosphorylation and associates with the Src homology 2 (SH2) domain-containing protein tyrosine phosphatase in stimulated platelets. For the first time, this study identified the potential antithrombotic drug target G6b-B in platelets. Similarly, also the receptor-like protein tyrosine phosphatase CD148 in platelets was identified as a regulator of platelet activation and an antithrombotic drug target [182].

The C-type lectin-like receptor 2 (CLEC-2) is a platelet transmembrane receptor that is able to modulate platelet activation during haemostasis, thrombosis and tumour metastasis [183,184]. A differential proteomic analysis of basal and rhodocytin-activated platelets from healthy volunteers was performed to study CLEC-2 signaling pathway, using 2D-DIGE, phosphotyrosine immunoprecipitations followed by 1D SDS-PAGE, and MS [115]. More than 100 proteins were differentially regulated after CLEC-2 platelet activation and several effectors of CLEC-2 signaling cascade were identified. 

The physiological and pathological roles of CLEC-2 were compared with GPVI, another antiplatelet drug target in thrombosis and thrombo-inflammatory disorders [185,186]. It was also demonstrated that CLEC-2, but not GPVI, is expressed in microparticles from activated platelets and it is not regulated by proteolysis, thus it can be used to monitor PMPs [187].

A proteomic approach has been also used to study glycolipid-enriched membrane domains, known as GEMs or lipid rafts, in human resting and stimulated platelets [188]. GEMs are rich in glycosphingolipids, cholesterol and saturated phospholipids, as well as specific membrane receptors and intracellular signaling proteins. They participate in platelet activation, primarily by GPVI, and are involved in signaling processes. GEMs isolation using non-ionic mild detergents is considered the main standard approach, but a wide variety of other detergents have been used. Both proteomic and lipidomic analysis demonstrated that Triton X100 1% is, in general, the most suitable detergent for lipid raft isolation [146], even if the choice is based on the study objectives, in particular, if the study is focused on the association between specific proteins and GEMs. García et al. identified more than 20 proteins in GEMs fractions of platelets in a basal state using nanoLC-MS/MS, including membrane proteins (e.g., flotillin-1 and GPIV) and signaling proteins (e.g., tyrosine-protein kinases) [189]. Several GEMs-associated platelet proteins have been also identified by MALDI-TOF MS, among which stomatin was the major GEM component of α-granular membrane, while flotillin-1 and -2 were extensively present in resting platelets but not in α-granules. Moreover, after platelet activation by thrombin, stomatin translocated and selectively accumulated in released microvesicles, while flotillins were depleted from microvesicles reaching a different subcellular localization [94].

Recently, the first proteomic comparative analysis of GEMs obtained from resting platelets and platelets activated through GPVI and CLEC-2 receptors was performed by Izquierdo et al. [161]. The membrane fractions were isolated by sucrose gradient ultracentrifugation and GEMs-enriched fractions were determined by Western blot, then only the fractions enriched in flotillin-1 and lacking integrin αIIb were analyzed and GEMs proteins isolated. A significant number of signaling proteins highly expressed in lipid rafts following platelet activation was quantitatively measured by nanoLC-MS/MS. Moreover, GEMs reorganization upon GPVI and CLEC-2 activation caused a massive loss in cytoskeletal proteins, which were highly associated with these domains in resting platelets.

### 3.3. Post-Translational Modifications of Platelet Proteins

It is well-known that proteins structure and function are finely regulated by a large number (about 400) of PTMs and many of them are operative also in platelets.

#### 3.3.1. Protein Phosphorylation in Platelets

The role of phosphorylation in platelets has been described during haemostasis, where binding of adhesive substrates to tyrosine-kinase-linked adhesion receptors and/or soluble agonists to G-protein coupled receptors (GPCRs) activates intracellular signaling processes involving phosphorylation and dephosphorylation, but also in pathological states [109]. Indeed, during atherogenesis or vascular inflammation activated platelets increase the surface expression and ligand affinity of the integrin αIIbβ3 via inside-out signaling. Different stimuli through GPCRs activate protein kinases leading to the phosphorylation of the cytoplasmic tail of the integrin αIIbβ3, regulating its function [190].

Proteomic studies gained a deeper insight into phosphorylation both in resting and activated platelets. The first studies were based on the immunoprecipitation of phosphorylated proteins with specific antibodies against phosphotyrosine, and protein separation by 1D or 2-DE followed by identification with MS [91,93,98,102,191]. Maguire et al. studied the phosphotyrosine proteome from both resting and thrombin-activated human platelets, using an immunoprecipitation method followed by 2-DE gel analysis and MALDI-TOF MS [93]. They could obtain a specific subproteome containing relatively low-abundance proteins and detected 67 proteins unique of the thrombin-activated platelet proteome. Similarly, García et al. performed a study on the proteome of CRP-activated platelets, focusing in detail on tyrosine phosphorylation activated through the GPVI signaling cascade, identifying novel platelet phosphoproteins including the transmembrane protein Gf6 [102].

**Figure 4:**
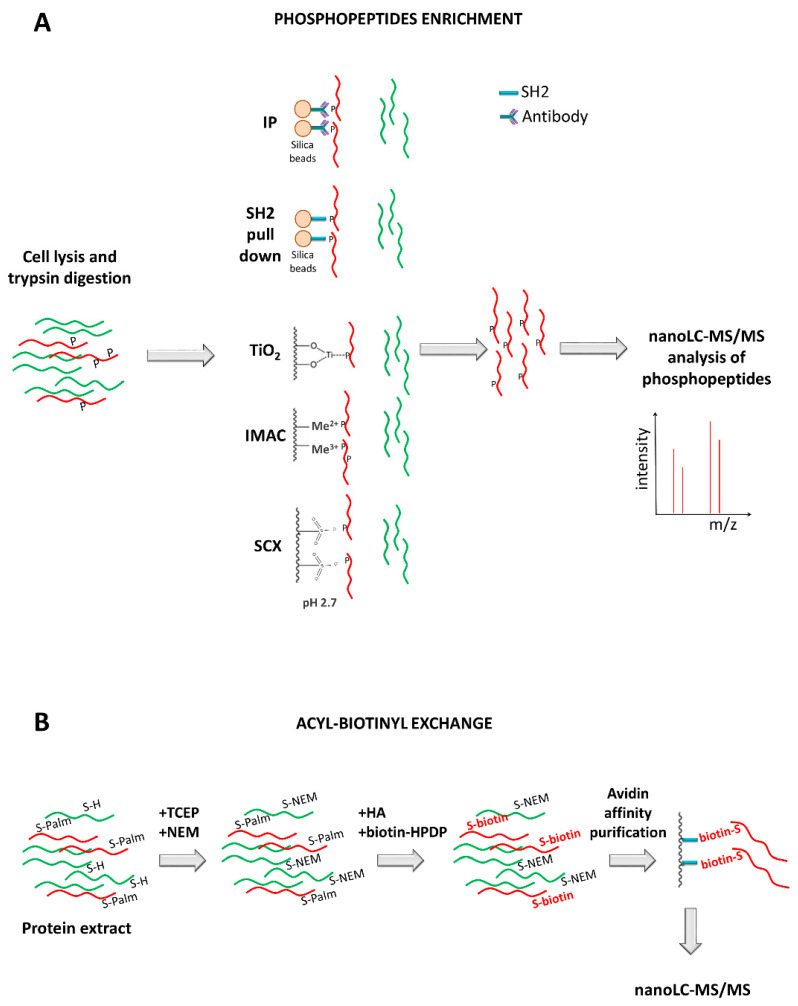
(**A**) Proteomic analysis of phosphoproteins. Typical workflows for phosphoprotein analysis include the lysis of cells and protein digestion, followed by phosphopeptide enrichment before LC-MS analysis. Different methods for phosphopeptides enrichment are depicted: immunoprecipitation (IP) with agarose beads coupled to an antibody against phosphotyrosine; pull-down with agarose beads coupled to SH2 domains with high affinity for pTyr; separation by titanium dioxide chromatography (TiO_2_); separation by immobilized metal-ion affinity chromatography (IMAC); strong cation exchange (SCX) chromatography. (**B**) Enrichment protocol for palmitoylated peptides. The acyl-biotinyl exchange protocol is based on protein denaturation, reduction with tris(2-carboxyethyl)phosphine (TCEP), and alkylation with N-ethylmaleimide (NEM) to block non-palmitoylated cysteines, followed by reaction with hydroxylamine (HA) and biotin-HPDP to replace palmitoyl groups with biotinyl groups. Thus, biotinylated proteins can be enriched by affinity purification and analyzed by proteomic approaches.

The advancement in the field of MS and the development of novel methods for phosphopeptide enrichment increased the number of identified entities, allowing a better clarification of protein network influenced by phosphorylation. Indeed, overcoming the problems associated with antibodies specificity, phosphopeptides can be separated using chromatographic approaches such as immobilized metal-ion affinity chromatography (IMAC), SCX chromatography, titanium dioxide chromatography (TiO_2_) or SH2 pull-down (Figure 4A). In particular, the high level of tolerance and robustness of TiO_2_ chromatography with most buffers used in biological experiments is the reason why it has become the method of choice in large-scale phosphoproteomic studies [192].

Zahedi et al. applied both IMAC and SCX and identified a total of 564 phosphopeptides in resting human platelets. Besides, they looked for specific protein kinase A and cGMP-dependent protein kinase phosphorylation sites, due to the presence of a common consensus sequence, and identified 12 unknown targets [109]. In another study again in resting platelets, Qureshi et al. characterized both the proteome and the phosphoproteome, creating a platelet protein–protein interaction network including the information derived from the phosphorylated proteins to introduce directionality in the protein–protein interaction graph [190].

Several studies have been also performed with stimulated platelets. The effects of oxidized phospholipids have been compared to thrombin stimulation using a comprehensive phosphoproteomic approach involving both immunoprecipitation of phosphotyrosines and affinity enrichment by IMAC or TiO_2_ before nanoLC-MS/M. The results of this study revealed a putative signaling pathway involving Src-family kinases, tyrosine-protein kinase SYK, and phospholipase C γ2 activated by oxidized phosphatidylcholine. These results provide novel insight into the mechanism of action of an important inducer of platelet hyperactivity and thrombosis in the presence of hyperlipidaemia or oxidative stress [132].

Beck et al. have addressed the time-dependent modulation of phosphorylation pattern in platelets treated with Iloprost to mimic the effects of endothelium-derived prostacyclin that represents an important physiological platelet inhibitor, acting through the activation of a cAMP/protein kinase A signaling cascade [133]. Using iTRAQ labelling and TiO_2_ enrichment, they followed the temporal alteration of the phosphorylation state of 2.700 phosphopeptides and identified 360 modulated peptides from proteins involved in Ca^2+^ and phosphatidylinositol signaling but also protein ubiquitination. More recently, the same group evaluated the phosphoproteome of platelets stimulated with ADP and the reversion induced by Iloprost. They provided temporal profiles of more than 4.000 phosphopeptides and evidenced a rapid induction of phosphorylation by ADP involving mainly proteins associated with actin and cytoskeleton remodelling. Besides, they identified some phosphorylation sites with a similar temporal behavior upon ADP or ADP + Iloprost and with an inverse regulation, which represent potential central players in platelet homeostasis and candidates for novel drug therapies [149].

#### 3.3.2. Protein Glycosylation in Platelets

Glycans can be produced by a set of competing glycosyltransferases resulting in the generation of structurally distinct glycans that lead to a condition known as site heterogeneity. Platelets express efficient glycosyltransferase machinery [193] and several studies evidenced a role for glycosylation in platelets activity. Indeed, most of the proteins involved in platelet adhesion are glycoproteins. Application of MS allowed better analysis of protein glycosylation and identification of glycosylation sites. The developed methods are based on enrichment of glycosylated proteins, removal of glycans, and identification by MS. The most used approach for glycan removal involves the use of PNGase, an enzyme that cleaves all type of glycans from proteins and converts asparagine to aspartic acid, leading to a characteristic mass shift of 1 Da [103]. On the other hand, different strategies have been proposed for the enrichment of N-glycosylated proteins based on affinity purification and have been applied to study glycoproteins in platelets. The first study by Lewandrowski et al. employed an enrichment with concanavalin A to trap N-glycan followed by PNGase and nanoLC-MS/MS, and lead to the identification of 70 glycopeptides corresponding to 41 glycoproteins including proteins involved in cell-cell communication [103]. The same group focused also the attention on platelet plasma membrane isolated by aqueous two-phase partitioning applying two different enrichment approaches: SCX prefractionation that takes advantage of the negative charge acquired by proteins with glycosylation by sialic acid [104], and electrostatic repulsion hydrophilic interaction chromatography (ERLIC) [110]. ERLIC allows the elution of glycopeptides in distinct fractions due to their different affinity for a weak anion exchange matrix overlaid with hydrophilic effects due to the use of a high amount of acetonitrile in the buffers. This strategy provided the identification of 125 glycopeptides from 66 platelets proteins, including important proteins involved in cell recognition or platelet adhesion [110]. Of note, a combination of different approaches has been applied to study the platelet N-glycosylation status in PMM2-congenital disorder of glycosylation (PMM2-CDG) patients, including immunoblotting, lectin-based enrichment of glycosylated proteins and expression of different membrane platelet N-glycoproteins by flow cytometry, as well as by platelet N-glycoproteome analysis with 2D-DIGE. Indeed, PMM2-CDG is an autosomal recessive disease with a multisystem presentation accompanied by an increased risk for thrombosis, which might be due to alteration of platelet function. However, despite a reduced sialic acid content in platelets membrane, this study revealed that the platelet proteome is not significantly altered in PMM2-CDG patients [134]. 

Recently, Toonstra et al. evaluated the effect of glycosylation on protein–protein interactions using a method based on the interaction of immobilized “bait”, specifically collagen, with soluble “prey” platelet glycoproteins with and without N-glycans, to elucidate the glycosylation roles in thrombus formation [159]. Results from this study demonstrated that major platelet adhesive receptors and proteins (i.e., coagulation-related proteins and extracellular matrix-binding proteins) required N-glycosylation to bind collagen suggesting the important role of this PTM in platelet functions.

O-glycosylation complexity limited the development of proteomic studies for its characterization in comparison to N-glycosylation. Nevertheless, several studies demonstrated the important role of this PTM in the haemostatic system and in particular, in platelet functions. Wang et al. generated transgenic mice with a targeted deletion in endothelial and hematopoietic cells of Cosmc, an essential chaperone that regulates protein O-glycosylation [194]. Using this model, they demonstrated that platelets exhibited a marked reduction of glycoprotein GPIB-IX-V complex function with loss of interactions with vWF, and a decreased agonist-mediated integrin αIIbβ3 activation reducing the interaction with fibrinogen. Therefore, O-glycans resulted to be essential for platelet biogenesis and regulation of their function in haemostasis. Recent developments in MS and glycan-enrichment methods, however, have enabled global studies of site-specific glycosylation. King et al. applied, to platelets, a dual vicia villosa agglutinin and peanut agglutinin lectin weak affinity chromatography for O-glycosylated proteins enrichment, coupled to nanoLC-MS/MS for identification and characterization of glycosylated peptides [150]. This is the first comprehensive analysis of O-glycosylated proteins that identified 1,123 O-glycosites, revealing that O-glycosylation is a ubiquitous modification occurring on extracellular proteins frequently close to proteases cleavage sites. Further, they performed in vitro peptide assays demonstrating that proteolysis of key haemostatic proteins can be inhibited by the presence of O-glycans. Despite this, it is clear that the effects of O-glycosylation are not only dependent on the PTM position but also the composition of glycans, the current technologies cannot analyze the glycosylation sites and the complex structure of glycans at the same time [150].

#### 3.3.3. Protein Palmitoylation in Platelets

Among lipid modifications, protein S-palmitoylation or S-acylation has been described in platelets. Palmitoylation of proteins is implicated in their association with membranes or specific lipid domains, such as rafts, but also in protein–protein interactions. S-acylation produces a strong membrane anchor, which specifies the membrane subdomain to which modified proteins are localized allowing their movement between cellular locations, even if it is not the primary membrane targeting signal [195].

For the first time, the role of protein palmitoylation in platelet activation and thrombus formation has been depicted by Sim et al. using acyl protein thioesterase 1 to depalmitoylate and blocking palmitoylation with a specific inhibitor, cerulein [196]. They demonstrated that cerulein inhibits platelet activation and aggregation induced by ADP, epinephrine, thrombin, collagen, CRP, and thrombin receptor activating peptide (SFLLRN). Moreover, they evidenced an in vivo reduction of platelet accumulation in thrombi generated by laser-induced injury of mouse cremaster arterioles. 

Proteomic approaches to study protein palmitoylation have been described thanks to the development of purification methods that selectively replace palmitoyl groups with affinity purification tags [197] or metabolic labelling with palmitic acid analogues. Acyl-biotin exchange is an enrichment protocol that involves blocking of free cysteines and release of palmitoyl thioester bonds using hydroxylamine (HA) before biotinylation of newly exposed cysteine thiol groups (Figure 4B). NanoLC-MS/MS can be used to identify labelled proteins purified using streptavidin resin. An alternative approach is based on metabolic labelling of cells with bio-orthogonal probes such as the palmitic acid analogue 17-ODYA, which is incorporated into proteins at sites of palmitoylation, and click-chemistry is used to specifically label 17-ODYA modified proteins with biotin before nanoLC-MS/MS analysis [198]. 

A proteomic approach based on acyl-biotin exchange has been applied to study human platelet palmitome in membranes of resting platelets identifying 215 palmitoylated proteins. Moreover, they validated the palmitoylation of triggering receptors expressed on myeloid cell (TREM)-like transcript-1 (TLT-1) using metabolic labelling with [^3^H]-palmitate, demonstrating that TLT-1 incorporated more palmitate after activation with thrombin and the specific modification site was cysteine 196. This study represents the first extensive characterization of this important modification in platelets [123].

#### 3.3.4. Platelet Ubiquitome

Ubiquitination is a PTM generally linked with proteins degradation by the proteasome or by lysosomes but it is also implicated in many other cellular processes, including intracellular trafficking, inflammatory signaling, regulation of protein–protein interactions and association with ubiquitin (Ub)-binding scaffolds, both in physiological and pathological conditions [199].

Proteomics studies performed on platelet proteome showed that platelets express all the components of the Ub ligase system, including deubiquitinases at high copy numbers [124], and that ubiquitination is involved in platelet function. Gupta et al. demonstrated that platelets have a functional ubiquitin/proteasome system that can be modulated by proteasome inhibitors, such as MG132 or bortezomib, with strong effects on platelet functions [135]. MG132 suppressed the formation of occlusive, platelet-rich thrombi in FeCl3-damaged carotid arteries, and reduced platelet aggregation, spreading and clot retraction. Moreover, microparticle shedding induced by thrombin, ADP and lipopolysaccharide was reduced by proteasome inhibition. They also demonstrated, by immunoblotting of platelet proteins with specific antibodies, that the ubiquitination system can be stimulated by thrombin and ADP, and more effectively by MG132 pre-treatment. To deeply characterize the role of ubiquitination, they captured ubiquitinated platelet proteins by sushi domain columns or chromatography and identified the cytoskeletal proteins filamin A and talin-1 as targets of ubiquitination that accumulate in the cytoplasm of cells when proteasome activity is diminished [135]. Subsequently, the same authors demonstrated that platelets contain active deubiquitinases. Using a general deubiquitinase inhibitor and a specific inhibitor of E1 ubiquitin-activating enzyme, they evidenced the reduction of occlusive thrombosis in vivo and an ex vivo regulation of platelet aggregation, adhesion, and activation, involving the modulation of signaling downstream of GPCRs and GPIV receptor for collagen [200]. 

Recently, Unsworth et al. compared the ubiquitome of human platelets in resting condition and stimulated with cross-linked CRP (CRP-XL), employing an MS-based method after the enrichment of tryptic peptides with antibodies directed against a tag of two glycine ligated to ubiquitinated lysines [160]. This approach allowed the identification of 1,634 ubiquitinated peptides, 925 of which resulted to be increased in response to CRP-XL. The identified ubiquitinated proteins include SYK, which is a known component of the GPIV signaling pathway, filamin and talin-1 and members of the Rab family involved in vesicle trafficking. They also confirmed these results using a different enrichment protocol based on Tandem Ubiquitin Binding Entities (TUBEs) that have a high affinity for ubiquitinated proteins. In conclusion, they demonstrated that ubiquitination is a regulatory component of signaling activated by collagen through the GPIV receptor. 

#### 3.3.5. Oxidative Stress and Platelets Protein Modification

Among the factors that can influence platelet function and thrombus formation, a relevant role is exerted by oxidative stress and, specifically, by the formation of superoxide and nitric oxide, as well as their metabolites [201]. Despite reactive oxygen species, originating from platelets or other vascular sources, to regulate normal platelet activation and aggregation, additional oxidative stress in certain settings may be prothrombotic, while reactive nitrogen species have been shown to inhibit platelet function [201]. An excess of oxidative stress can alter protein structure and function as the result of protein carbonylation through direct oxidation of specific amino acids or formation of specific protein adducts with lipid peroxidation products, such as α,β-unsaturated aldehydes (i.e., 4-hydroxynonenal, 4-HNE) [202]. Indeed, increased carbonylation of platelet proteins occurs in the presence of exogenous oxidative stress, thrombin stimulation and progression of ageing and diabetes [203]. Considering that platelet aggregation induced by thrombin can be reduced by 4-HNE, Ravi et al. studied the alteration of platelet metabolome and proteome in response to 4-HNE [145]. Of note, to determine the specific protein targets of 4-HNE in platelets, they used an analogue of HNE, which contains a terminal alkyne group that can be conjugated to a biotin tag using click chemistry, allowing for affinity enrichment of HNE-adducts using avidin resin and identification by MS. They found that 4-HNE modified 72 proteins with key roles in platelet activation, including proteins involved in metabolism, adhesion, cytoskeletal reorganization, aggregation, vesicular transport, protein folding, antioxidant proteins, and small GTPases [145]. The role of oxidative stress-induced protein modification has been also extensively studied in the context of platelet storage where platelets concentrates treated with pathogen inactivation technologies exhibit an increase in oxidative stress [204]. Higher levels of carbonylation have been detected in platelet concentrates treated with Mirasol, a proprietary pathogen reduction technology system [205].

It is well known that nitric oxide is an important signaling molecule able to regulate platelet function and induce protein PTM, but a comprehensive analysis of nitric oxide-modified platelets proteins is still lacking. The role of S-nitrosylation has been demonstrated in the conformational changes that regulate the activation/deactivation of platelet-specific integrin αIIbβ3 [206], as well as in the nitric oxide-induced inhibition of dense granules, lysosomal granules and α-granules secretion mediated by *N*-ethylmaleimide-sensitive factor (NSF) [207]. Indeed, several S-nitrosylated proteins have been identified under basal conditions and after treatment with exogenous nitric oxide-donors [208]. Tyrosine nitration of α-actinin has been suggested as a key modulatory mechanism controlling platelet adhesion [209], and nitration of platelet vasodilator-stimulated phosphoprotein is an important mechanism in the regulation of platelet shape change involved in the inhibition of platelets aggregation [210]. Nitroxyl (HNO/NO-) is an alternative redox form of nitric oxide, considered due to its effects on failing hearts, with potential effects on platelet functions, including inhibition of platelet aggregation [211]. Hoffman et al. applied a global proteomic analysis of platelets treated with an HNO donor to discover the protein targets of this modification following, by MS, the formation of a sulfonamide modification giving a specific mass shift of 65 Da. This approach led to the identification of 10 dose-dependently modified proteins in platelets metabolism, cytoskeletal rearrangement and signal transduction [112].

### 3.4. Proteomic Study of Antiplatelet Agent Effects

The use of proteomic technologies encouraged the search for a protein profile indicative of the response of a drug. Numerous studies have shown that human platelet protein profiles can be successfully used to evaluate the drug effects and several antiplatelet agents have been employed. Despite the availability of several drugs and the widespread use of dual antiplatelet therapy, the pharmacological response of patients is highly variable. Many patients do not respond or have a poor response to specific antiplatelet drugs, and continue to experience recurrent thrombotic events [212].

Platelet proteomics has been applied, for example, in studying the effects of ASA. Platelets were stimulated using three different agonists in the presence and absence of ASA, and a significant reduction of platelet aggregation was found with ASA and all agonists. Different releasate profiles were observed using SDS-PAGE and MS, and ASA caused a general decrease in the amount of protein released independently of the agonist used [85]. Later, significantly changes were observed between stable coronary ischaemic ASA-resistant and ASA-sensitive patients using 2-DE and MALDI-TOF/TOF MS, including proteins involved in energetic metabolism, cytoskeleton, oxidative stress and cell survival [120]. Moreover, recently, the platelet glycoproteome was studied using iTRAQ labelling followed by MS in resting and stimulated healthy platelets with and without ASA treatment [152]. Several identified glycoproteins were significantly differentially regulated in platelet lysates from ASA-treated versus untreated platelets after activation with collagen. In particular, metallopeptidase inhibitor 1 was the highest affected glycoprotein by ASA treatment. 

Platelet proteome was also investigated in patients with stable angina undergoing percutaneous coronary intervention during the onset of clopidogrel therapy [126]. Aiming to evaluate platelet reactivity and clopidogrel response, 2DE and MALDI-TOF/TOF MS were applied, providing the identification of some differentially expressed proteins. Using the same techniques, another group compared the effect of dual antiplatelet therapy (ASA and clopidogrel) with ASA alone on platelet proteomes of type 2 diabetic patients with stable coronary ischemia [127]. Only a small fraction of proteins linked to cytoskeleton, contractile system, energetic metabolism, inflammation, and antioxidant system was differentially regulated after dual therapy, thus suggesting that the combination of clopidogrel and ASA did not increase the platelet inhibition in patients enrolled in this study. Moreover, plasma levels of PF4, which is an index of platelet activation in vivo, were unchanged between the two groups. 

However, dual antiplatelet therapy with ASA and clopidogrel or other ADP antagonist is widely used in several pathological conditions, such as ACS, peripheral arterial disease, and in patients with risk of myocardial infarction and ischaemic stroke [212,213].

Another inhibitor of the ADP receptor P2Y_12_ is Iloprost, which is a second-generation structural analogue of prostacyclin able to inhibit thrombin- and collagen-induced aggregation of human platelets. Inhibitors of the ADP receptor P2Y_12_ are widely used in the prevention of cardiovascular injury and as secondary prophylaxis after cardiovascular events; however, extensive studies are still needed to better clarify their molecular mechanisms due to high variability in drug efficacy. As an example, temporal quantitative phosphoproteomics was performed in human platelets by MS after stimulation with ADP and ADP + Iloprost [149]. For a set of phosphorylation sites, the authors showed an inverse regulation by inhibition with Iloprost, highlighting their role as modulators of platelet homeostasis and potential candidates for monitoring platelet activation. These selected phosphorylation sites were quantified by PRM with high precision and accuracy.

Marcone et al. applied a gel-free nanoLC-MS/MS approach to study proteomic signatures of three different antiplatelet drugs: ASA, a P2Y_12_ antagonist, and a PAR-1 antagonist [212]. The response to antiplatelet drugs was evaluated in human healthy platelet releasates following stimulation with thrombin, and the effects of these drugs were recognizable when used alone or in combination. The study showed evident drug signatures with differences between the three antiplatelet agents, and combinations of two or three drugs revealed distinctive platelet protein profiles. 

Sarpogrelate is another attractive antiplatelet agent, an antagonist of 5-hydroxytryptamine receptors, and it has been widely used in the treatment of arterial occlusive diseases because it inhibits serotonin-induced platelet aggregation, vasoconstriction, and vascular smooth muscle proliferation. A comprehensive proteome profiling of platelets from subjects before and after sarpogrelate administration has been performed by nanoLC-MS/MS [147]. Platelet inhibition with sarpogrelate showed several differently regulated proteins primarily involved in cell activation, coagulation, and vesicle-mediated transport. The authors created a panel of proteins that could represent the effects of sarpogrelate on platelets and discriminate samples before and after administration.

### 3.5. Targeted Proteome on Platelets

Over recent years, targeted quantification methods have been increasingly used for robust and accurate quantification of selected proteins across many samples and replicates. As mentioned before, targeted proteomics is usually performed by SRM/MRM or PRM MS, providing the monitoring and quantification of a limited number of pre-defined peptides per analysis. For absolute quantification, isotope-labelled standards matching the sequence of the endogenous targeted peptides are added to the sample before any preprocessing. Moreover, fractionation strategies for further improving sensitivity are usually applied before the MS analysis. 

Recently, targeted PRM analysis has been used to absolute quantify promising CVDs biomarkers in human platelets for the study of platelet signaling, secretion, and aggregation responses [153]. The authors measured the absolute concentration of a subset of peptides and corresponding proteins with high accuracy and precision via an internal calibration curve in a reference sample of platelets pooled from healthy donors. These proteins were related to platelet dysfunction or activation processes and potentially involved in the progression of thrombotic conditions. 

Targeted quantitative proteomics was also used to investigate the molecular mechanisms and changes in platelets from Scott syndrome patients [144]. The authors initially performed a global proteome and phosphoproteome analysis of stimulated Scott and control platelets using iTRAQ stable isotope labelling in combination with TiO_2_ enrichment and high pH reversed phase fractionation. Then, they used high-resolution PRM to quantify and validate selected proteins that were differently expressed in Scott platelets, confirming, in particular, the absence of anoctamin-6 and the upregulation of aquaporin-1.

A stable isotope dilution immunopurification 2D nanoUHPLC-PRM MS was also applied in a proteomic study for analyzing the protein frataxin in platelets from Friedreich’s ataxia patients [158]. This highly sensitive and specific approach allowed to measure frataxin in platelets with no overlap between controls and patients, thus the assay showed very high discriminative power for the two studied populations.

Another strategy is the application of plasma QconCAT-based targeted proteomics, which allows the multiplex absolute quantification of hundreds of proteins in a single LC-MRM MS run [214]. This approach was applied to monitor storage and gender-dependent proteomic changes in blood samples, and approximately 100 proteins were absolutely quantified in apheresis platelet concentrate supernatants from male and female donors at diverse storage days [141]. The results showed that samples from female donors were less stable. Indeed, several proteins had an increased concentration in female platelet supernatants and their levels changed in a storage-dependent mode. This study demonstrated that QconCAT-based MRM strategy could be a promising approach in the field of transfusion-medicine.

Moreover, a targeted quantitative proteomic approach using LC-MRM MS has been successfully used to monitor in parallel small GTPase isoforms at different time points from human platelets in response to several agonists [138].

## 4. Conclusions

Over the years, the developments in the field of platelet biology have provided important insights into platelet structure, multiple functions, heterogeneity, signaling and interactions, emphasizing their central role in the pathophysiology of a wide variety of diseases, beyond the disorders of coagulation. In particular, proteomics has emerged as a powerful tool for characterizing the multifunctional nature of platelets both in health and disease conditions. 

Great improvements in the knowledge of platelet proteome have been achieved thanks to the high-throughput, efficiency, and sensitivity of proteomics techniques, allowing the identification of a considerable number of new proteins and their modifications. For example, G6b-B isoform and the receptor-like protein tyrosine phosphatase CD148 were identified as potential antithrombotic drug targets in platelets. Proteomics on platelets also helped to clarify the role of lipid rafts in platelet activation encouraging, in the near future, further biochemical and functional studies of these membrane microdomains in platelets [167].

Due to improved sample preparation protocols and increased experience in MS-based identification and quantification, as well as more sophisticated bioinformatics software, proteomic studies have yielded additional information about platelet biology and their role in diseases. Recent advances in proteomics helped to identify potential platelet biomarkers involved in the progression of CVDs, cancer and other pathologies, and novel antiplatelet therapeutic targets. 

**Figure 5:**
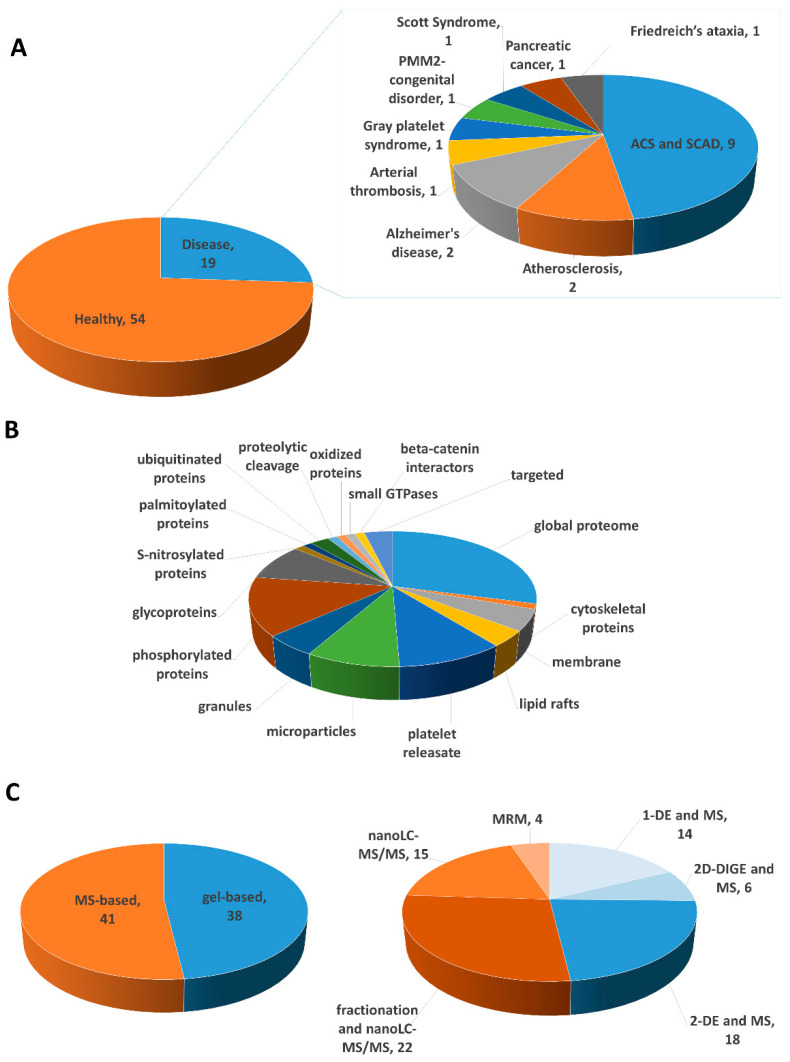
A summary of the proteomic studies on platelets: (**A**) Distribution of proteomic studies according to the type of sample: platelets from healthy subjects or patients; (**B**) distribution of proteomic studies according to the investigated compartment; (**C**) distribution of proteomic studies according to the employed technics.

The majority of the proteomics studies described in this review has been performed on platelets from healthy subjects, to characterize their composition or specific signaling pathways (Figure 5). Nevertheless, several studies successfully helped to clarify the molecular mechanisms involved in changes in platelet proteins that might reflect a specific pathological status. For instance, a persistent platelet hyperactivity after the acute thrombotic event has been described following a perturbation in the cytoskeletal organization [109], or an increase of some proteins in intracoronary platelets at the culprit vessel has been reported in STEMI patients, driving the attention towards specific signaling pathways as a source of relevant biomarkers or antiplatelet targets [114]. In recent years, platelet proteomics has seen progress not only in the cardiovascular field, but also in cancer [115] and neurological disorders [116] highlighting that the platelet proteome can be considered for potential biomarkers of both tumour presence and disease status. Indeed, a tumour-type dependency of platelet changes was found due to differences in cancer cell secretome and tumour localization, as well as new differentially expressed proteins in platelets from AD patients involved in several cytoskeletal-mediated pathways, inflammation and immune functions.

Proteomics has also contributed to monitoring platelet proteome changes during a disease follow-up, identifying the main receptors and signaling proteins involved. Some of them could represent potential antithrombotic targets, such as a higher activation of the collagen receptor GPVI signaling cascade and sarcoma protooncogene tyrosine-protein kinase pathway in STEMI patients [112].

Although, in recent decades, proteomic studies on platelets significantly increased, providing promising results, some important issues are still unresolved for the application of platelet biomarkers in clinical practice. Most of the proteomic studies on platelets carried out so far, have been performed on a very small sample size without a validation phase on an independent casistic that is useful for a confirmation of the specificity, sensitivity and robustness of the potential identified biomarkers. Large cohort samples are needed, alongside the standardization of sample preparation and data processing, for large-scale clinical application of platelet proteomics.

Together with the significant progress of proteomic technologies, standardized protocols of platelet preparation, activation and protein isolation are essential for the future application in clinical research of the platelet analysis as biomarkers. 

Considering that platelets are involved in pathologies of various nature and many disorders are likely attributable to alterations in protein expression levels and quantification, the study of platelet protein composition, interactions with other cell molecules, and PTMs would help the understanding of pathological mechanisms as well as the development of specific therapeutic interventions.

Further, a wide range of platelet proteins have been characterized for potential use in disease identification and therapeutic treatment.

In the near future, the vast amount of proteomic data, especially from quantitative studies, combined with other strategies such as metabolomics, transcriptomics, and bioinformatics, may contribute improving the knowledge of signaling networks and nodes underlying the platelet response to several pathophysiological pathways. Thereby, it will be possible in future to develop new antiplatelet therapies that are directed against specific platelet responses, subpopulations, interactions, or priming conditions, with a reduced threat of adverse effects. It will be important to evaluate the individual variability in the proteome of healthy subjects and patients with the aim of developing a personalized and precision medicine. The integration of biochemical and functional data with the quantitative values of platelet proteins would provide more detailed information for the development of new pharmacologic targets that might be applied in the precision medicine.

## Figures and Tables

**Table 1:** Summary of the proteomics studies on platelets.

Year	Disease	Sample Size	Stimulus/Treatment	Compartment	Total Identified Proteins *	Proteomic Method	Verification Methods	Validation on Independent Cohort	Reference
2000	healthy	n/a		global proteome	186	2-DE and MALDI-TOF			[91]
				phosphorylated proteins	28	2-DE and MALDI-TOF			[91]
2000	healthy	n/a	thrombin	cytoskeletal proteins	n/a (27)	2-DE and MALDI-TOF/TOF			[92]
2002	healthy	n/a	thrombin	Phosphorylated proteins	n/a (67)	2-DE and MALDI-TOF	IB		[93]
2002	healthy	n/a		lipid rafts	n/a (3)	1-DE and MALDI-TOF			[94]
2003	healthy	n/a		cytosolic and membrane fraction	264	COFRADIC			[95]
2004	healthy	n/a		global proteome	760	2-DE and nanoLC-MS/MS			[96]
2004	healthy	n/a		global proteome	163	COFRADIC			[97]
2004	healthy	n/a	TRAP	global proteome	~1069 (41)	2-DE and nanoLC-MS/MS	IB		[98]
2004	atherosclerosis	n/a	thrombin	platelet releasate	81 (9)	2-DE and MALDI-TOF; MudPIT	confocal microscopy; IHC		[99]
2005	healthy	n/a	ADP	microparticles	578	1-DE and nanoLC-MS/MS			[100]
2005	healthy	n/a		membranes	297	1-DE and nanoLC-MS/MS	IB		[101]
2006	healthy	n/a	collagen related peptide	phosphorylated proteins and global proteome	~1800 (117)	2-DE and LC- MS/MS	IB		[102]
2006	healthy	n/a		N-glycoproteins	41	ConA trapping and nanoLC-MS/MS			[103]
2007	healthy	n/a		N-glycoproteins in plasma membranes	79	SCX and nanoLC-MS/MS			[104]
2007	atherosclerosis	n/a healthy subjects; 3 patients with delta storage pool disease		dense granules	n/a (40)	2-DE and MALDI TOF/TOF or nanoLC-MS/MS	IB; IHC		[105]
2007	healthy	n/a		α-granule	300	1-DE and nanoLC-MS/MS	microscopy		[106]
2007	healthy	n/a		membranes	136	nanoLC-MS/MS	IB		[107]
2007	healthy	3	ASA and ADP, collagen or TRAP	platelet releasate	146	1-DE and nano-LC-MS/MS	IB; antibody arrays		[85]
2008	arterial thrombosis	29 patients; 24 controls		global proteome	n/a (3)	2-DE and MALDI-TOF	IB		[108]
2008	healthy	n/a		Phosphorylated proteins	270	IMAC, SCX and nanoLC-MS/MS	IB		[109]
2008	healthy	n/a		N-glycoproteins	66	ERLIC and nanoLC-MS/MS			[110]
2009	healthy	3	TRAP	platelet releasate	325	1-DE and LC-MS/MS	n/a		[111]
2009	healthy	10		phosphorylated proteins	262	1-DE, IMAC and nanoLC-MS/MS			[111]
				global proteome	1507	nanoLC-MS/MS			
2009	healthy	n/a	HNO donors	S-nitrosylation	n/a (21)	1-DE and LC-MS/MS	LC-MRM		[112]
2009	healthy	n/a	thrombin and collagen	microparticles	546	gel filtration and 2D-LC-MS/MS			[113]
2009	healthy	n/a		membranes	1282	COFRADIC, 1-DE, SCX and nanoLC-MS/MS			[114]
2009	healthy	n/a	rhodocytin	phosphorylated proteins	83	1-DE and LC-MS/MS	IB		[115]
				global protein	2000(73)	2D-DIGE and MALDI- TOF/TOF	IB		[115]
2010	ACS and SCAD	12 SCAD patients; 14 NSTEMI patients; 10 healthy subjects		global proteome	~400 (6)	2-DE and nanoLC-MS/MS	IB; enzymatic assays		[116]
2010	ACS and SCAD	18 NSTEMI patients; 10 SCAD patients		global proteome	n/a (40)	2-DE and MALDI-TOF/TOF	IB		[117]
2010	healthy	7	mAb HGP4C9	platelet releasate	n/a (13)	2D-DIGE and LC-MS/MS	IB		[118]
2010	Gray platelet syndrome	1 patients		α-granule	n/a	1-DE and LC-MS/MS	microscopy		[119]
2010	SCAD	51 SCAD	ASA	global proteome	n/a (~17)	2-DE and MALDI-TOF/TOF	IB; enzymatic assays		[120]
2011	ACS and SCAD	11 STEMI patients; 15 SCAD patients		global proteome	n/a (42)	2-DE and MALDI-TOF/TOF	IB	10 healthy volunteers	[121]
2011	ACS and SCAD	16 ACS patients; 26 SCAD patients		global proteome	n/a (22)	2-DE and MALDI-MS/MS	IB; enzymatic assays		[122]
2011	healthy	n/a		palmitoylated proteins	215	acyl-biotin exchange and nanoLC-MS/MS	metabolic labeling		[123]
2012	healthy	4		global proteome	4000	nanoLC-MS/MS			[124]
2012	healthy	5	thrombin and shear stress	microparticles	n/a (26)	2-DE and MALDI TOF/TOF	IB		[125]
2012	SCAD	20 SCAD patients undergoing PCI	clopidogrel	global proteome	n/a (18)	2-DE and MALDI-TOF/TOF	IB		[126]
2012	SCAD	57 SCAD patients with type 2 diabetes	ASA and clopidogrel	global proteome	n/a (8)	2-DE and MALDI-TOF/TOF			[127]
2013	healthy	3	thrombin and collagen	platelet releasate	4116 (124)	2D nanoLC-MS/MS			[128]
2013	healthy	n/a	ADP	microparticles	600	nanoLC-MS/MS			[129]
2013	Alzheimer’s disease	7 control subjects; 7 AD patients		membranes	1009 (144)	nanoLC-MS/MS	IB		[130]
2014	healthy	3	PAR-1 and PAR-4 agonists	platelet releasate	2296 (93)	SCX fractionation and nanoLC-MS/MS			[131]
2014	healthy		thrombin and oxidized phospholipids	Phosphorylated proteins	418(115by oxPC and 181 by thrombin)	IMAC, SCX, TiO_2_ and nanoLC-MS/MS	IB; FC		[132]
2014	healthy	4	Iloprost	Phosphorylated proteins	~2700(299)	TiO_2_ and nanoLC-MS/MS	IB		[133]
2014	PMM2-congenital disorder	11 healthy subjects; 6 patients		N-glycoproteins	n/a (12)	2D-DIGE and MALDI TOF/TOF			[134]
2014	healthy	n/a		ubiquitinated proteins	n/a	affinity purification and nanoLC-MS/MS	IB		[135]
2014	healthy	n/a	Ca^2+^ ionophore, thrombin and collagen	microparticles	~200	1-DE and nanoLC-MS/MS			[136]
2014	healthy	n/a		granules	827	nanoLC-MS/MS	IF		[137]
2015	healthy	n/a	thrombin, ADP, collagen, alkyl-LPA	small GTPases	12	LC-MRM MS	IB		[138]
2015	healthy	n/a	thrombin and collagen	platelet releasate	n/a (37)	2D-DIGE and nanoLC-MS/MS	IB		[139]
2015	healthy	n/a	ADP, thrombin and collagen	microparticles	3000	nanoLC-MS/MS			[140]
2015	healthy	6		targeted	139	QCONCAT LC-MRM			[141]
2016	ACS and SCAD	10 STEMI	intracoronary and peripheral platelets	global proteome	~1300 (16)	2D- DIGE and MALDI-TOF/TOF	IB		[142]
2016	ACS and SCAD	5 STEMI; 5 SCAD	collagen related peptide	phosphorylated proteins	n/a (26)	1-DE and nanoLC-MS/MS	IB	14 STEMI e 11 SCAD	[143]
2016	Scott Syndrome	4 healthy subjects; 1 patients	thrombin, thrombin/convulxin, ionomycin	phosphorylated proteins	709	TiO_2_ and nanoLC-MS/MS	LC-PRM, IB; FC		[144]
				proteolytic cleavage	375	ChaFRADIC and nanoLC-MS/MS	LC-PRM, IB; FC		[144]
				global proteome	2278 (134)	nanoLC-MS/MS	LC-PRM, IB; FC		[144]
2016	healthy	n/a	4-HNE	oxidized proteins	72	affinity purification and nanoLC-MS/MS			[145]
2016	healthy	n/a		lipid rafts	822	1-DE and MALDI-TOF/TOF			[146]
2016	healthy	18 healthy subjects	Sarpogrelate	global proteome	5423 (499)	nanoLC-MS/MS	IB	5 subjects	[147]
2017	healthy	10		global proteome	3036	nanoLC-MS/MS	FC		[148]
2017	healthy	n/a	ADP and Iloprost	phosphorylated proteins	1600 (302 by ADP)	TiO_2_ and nanoLC-MS/MS	IB; LC-PRM		[149]
2017	healthy	4		O-glycoproteins	649	affinity purification and nanoLC-MS/MS	in vitro peptide assay		[150]
2017	healthy	3	thrombin	microparticles	400	nanoLC-MS/MS			[151]
2017	healthy	10	collagen and ASA	glycoproteins	424 (21)	nanoLC-MS/MS	ELISA		[152]
				global proteome	1532 (15)	nanoLC-MS/MS	ELISA		[152]
2017	healthy	5		targeted	99	LC-PRM			[153]
2018	pancreatic cancer	12 patients; 11 controls		global proteome	4384 (85)	1-DE and nanoLC-MS/MS	-		[154]
2018	Alzheimer’s disease	115 AD patients; 49 controls		global proteome	n/a (22)	2D- DIGE and MALDI-TOF/TOF	IB		[155]
2018	healthy	n/a	TRAP	beta-catenin interactors	9	IP and nanoLC-MS/MS	IB		[156]
2018	healthy	32	thrombin	platelet releasate, exosome enriched	277	LC-MS/MS			[157]
2018	Friedreich’s ataxia	7 patients; 7 healthy subjects		targeted	1	LC-PRM			[158]
2019	healthy	n/a		N-glycoproteins		affinity purification and nanoLC-MS/MS			[159]
2019	healthy	n/a	collagen-related peptide	ubiquitinated proteins	691	affinity purification and nanoLC-MS/MS			[160]
2019	healthy	n/a	collagen-related peptide or rhodocytin	lipid rafts	447	nanoLC-MS/MS			[161]

* differentially expressed proteins are reported between brackets; 1-DE, 1-dimensional electrophoresis; 2D-DIGE, 2- dimensional differential gel electrophoresis; 2-DE, 2-dimensional electrophoresis; 4-HNE, 4-hydroxynonenale; ACS, acute coronary syndrome; AD, Alzheimer’s disease; ASA, acetyl salicylic acid; ChaFRADIC, charge-based fractional diagonal chromatography; COFRADIC, combined fractional diagonal chromatography; IB, immunoblotting; IHC, immunohistochemistry; IMAC, Immobilized metal affinity chromatography; IP, immunoprecipitation; LC, liquid chromatography; MALDI, matrix-assisted laser desorption ionization; MRM, multiple reaction monitoring; MS, mass spectrometry; MudPIT, Multidimensional Protein Identification Technology; NSTEMI, non ST-elevation myocardial infarction; PCI, Percutaneous coronary intervention; PRM, parallel reaction monitoring; SCAD, stable coronary artery disease; SCX, strong cation exchange; STEMI, ST-elevation myocardial infarction; TOF, time of flight.
